# Solvation dynamics in polar solvents and imidazolium ionic liquids: failure of linear response approximations†
†Electronic supplementary information (ESI) available: Higher order and partial correlation functions, corrections to Gaussian statistics and structure analysis for all systems. See DOI: 10.1039/c7cp07052g


**DOI:** 10.1039/c7cp07052g

**Published:** 2018-01-29

**Authors:** Esther Heid, Christian Schröder

**Affiliations:** a University of Vienna , Faculty of Chemistry , Department of Computational Biological Chemistry , Währingerstraße 19 , A-1090 Vienna , Austria . Email: christian.schroeder@univie.ac.at ; Tel: +43 14277 52711

## Abstract

This study presents the large scale computer simulations of two common fluorophores, *N*-methyl-6-oxyquinolinium betaine and coumarin 153, in five polar or ionic solvents. The validity of linear response approximations to calculate the time-dependent Stokes shift is evaluated in each system. In most studied systems linear response theory fails. In ionic liquids the magnitude of the overall response is largely overestimated, and linear response theory is not able to capture the individual contributions of cations and anions. In polar liquids, the timescales of solvation dynamics are often not correctly reproduced. These observations are complemented by a detailed analysis of Gaussian statistics including higher order correlation functions, variance of the energy gap distribution and its time evolution. The analysis of higher order correlation functions was found to be not suitable to predict a failure of linear response theory. Further analysis of radial distribution functions and hydrogen bonds in the ground and excited state, as well as the time evolution of the number of hydrogen bonds after solute excitation reveal an influence of solvent structure in some of the studied systems.

## Abstract 2

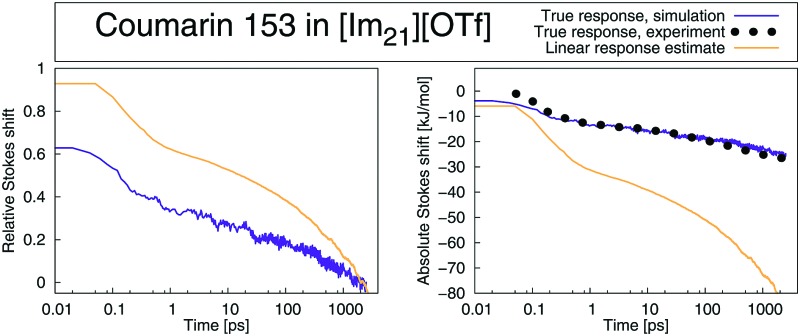
Large scale computer simulations of different fluorophore-solvent systems reveal when and why linear response theory applies to time-dependent fluorescence measurements.

## 1 Introduction

The time-dependent Stokes shift (TDSS) describes the rate of solvent reorganization after an electric perturbation inflicted by the excitation of an immersed chromophore. The TDSS has been extensively used to investigate the solvation dynamics of polar solvents over the last decades.^1^–^16^ With the development of ionic liquids as interesting new solvents for diverse reactions, the TDSS has also become a powerful tool to examine the electrostatic solvation in ionic, non-aqueous media.^17^–^29^ In contrast to conventional solvents such as water and alcohols, the solvation response in ionic liquids is extremely slow, as it scales with viscosity.^25^ A number of computer simulations have been conducted to examine the interesting solvation behavior of ionic liquids.^22^,^25^,^30^–^32^ It was shown that the solvation energies of ionic liquids resemble those in conventional solvents and most of the solvation energy stems from anion movement. However, the computer simulation of solvation dynamics is often realized *via* invocation of linear response theory (LRT), which may not be valid for arbitrary solute–solvent combinations. Within linear response theory, the true solvent reorganization after the excitation of a solute is resembled by the extrapolation of the energy gap fluctuations between the ground and excited state. This approximation may save a lot of computation time, but at the cost of being valid only in systems where either the perturbation is small, or the energy gap fluctuations are Gaussian.^4^,^33^–^35^ We recently showed that LRT fails for systems where the energy gap is locally Gaussian, but non-stationary (changes with time), or if large changes in solvent structure occur after perturbation, both leading to a globally non-gaussian distribution.^36^

In literature, the TDSS is often calculated only *via* LRT^27^,^30^,^37^–^46^ and the true nonequilibrium response of a system remains unknown. This is especially true if computationally expensive all-atomic and polarizable ionic liquid forcefields are used. Ref. 32 (or the earlier work ref. 31) is frequently used to justify the use of linear response theory in solvation dynamics of ionic liquids.^30^,^37^,^39^–^43^ In this work of Kim and coworkers a set of 400 nonequilibrium simulations extending to 2 ps is used to describe the solvation dynamics of artificial solutes in two imidazolium ionic liquids, and compared to equilibrium simulations invoking linear response theory.^32^ Although the authors argue that linear response holds reasonable well in their system, the agreement between the Stokes shift from their equilibrium and nonequilibrium simulations seems to be unsatisfactory, and the integral timescales differ considerably. Furthermore, ionic liquid dynamics, extending to nanoseconds, cannot be simulated accurately on such a short timescale. Although this work comprises valuable insights into ionic liquid solvation, it cannot be invoked to justify the choice of LRT for solvation dynamics in ionic liquids without further verification. A more recent study of Maroncelli and coworkers revealed the failure of linear response for the solvation dynamics of small solutes in ionic liquids.^25^ Current advances in technology opened up the possibility of calculating large scale nonequilibrium simulations, so that the validity of LRT in these systems can now directly be assessed. This study therefore focuses on the validity of the linear response approximation both in polar and ionic liquids using fully atomistic, flexible and polarizable force fields by direct comparison to nonequilibrium simulations, as well as experimental data. Real chromophores are employed as solutes, as the solvation response depends strongly on the nature of solute. Thus, the commonly used artificial solutes can only give general information. We study *N*-methyl-6-oxyquinolinium betaine (MQ) and coumarin 153 (C153) as the former lowers its dipole moment upon excitation, whereas the latter strengthens it, so that the validity of linear response can be tested for both an increase and decrease of the dipole moment during excitation. Furthermore the ability of partial correlation functions to depict cationic and anionic contributions is assessed. Previously, different findings were reported on the predominant role of cations,^47^ anions^32^ or both^30^,^48^,^49^ for the short-time response, where some of the conclusions were drawn from equilibrium simulations. These results are complemented by an analysis of higher-order correlation functions and their ability to predict LRT validity,^35^,^50^ solvent structure *via* radial distribution functions and coordination numbers, as well as a time-evolution of the spectral width. Therefore, this work gives valuable insight into when and why linear response is applicable in real systems.

## 2 Theory

The following equations only comprise the most important relations of linear response theory, which will be employed in this study. The derivation of these relations, as well as a more detailed description, can be found in literature.^4^,^33^–^36^

Experimentally, a fluorescent molecular probe is introduced to the solvent of interest, excited by a laser beam, and its subsequent time-dependent change in fluorescence frequency monitored. The Stokes shift relaxation function can then be calculated as1
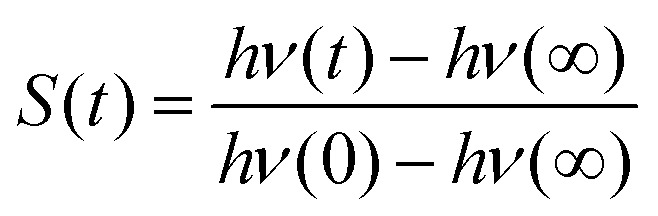
which is simply the normalized change in energy. Computationally, *S*(*t*) can be evaluated as the average change in interaction energy Δ*U*2
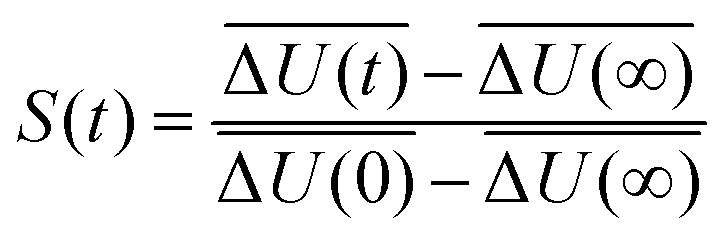
after excitation of a suitable chromophore. This procedure requires the actual excitation of the solute, so that an excited state force field is needed, as well as averaging over hundreds of different conformations. To avoid this computationally expensive method, often linear response theory (LRT) is invoked instead. Conventional LRT links the energy fluctuations δΔ*U*(*t*) = Δ*U*(*t*) – Δ*U* on the unperturbed energy surface (the equilibrated ground state) to *S*(*t*) *via*3
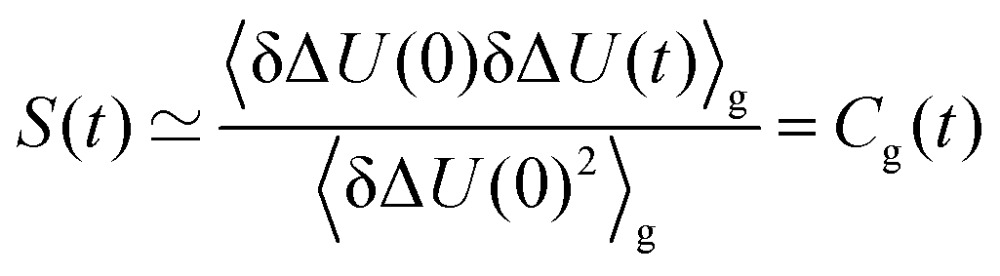
where we assume that the perturbation is small on a scale of *k*_B_*T*. Alternatively, the energy fluctuations on the perturbed energy surface (the equilibrated excited state) can be linked to *S*(*t*) *via*4
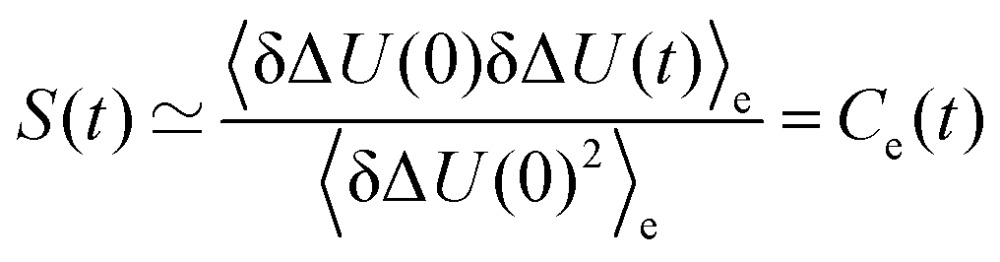
In both cases, the large number of nonequilibrium simulations needed for eqn (2) are replaced by a single equilibrium simulation on the ground or excited state. The latter relation is true even for large perturbations, if the system exhibits Gaussian statistics.^34^,^35^ Then, higher order correlation functions which are treated as zero in conventional LRT, are taken into account, as they are multiples of δΔ*U*(0)δΔ*U*(*t*)_e_ (odd-numbered), or zero (even-numbered):^51^5
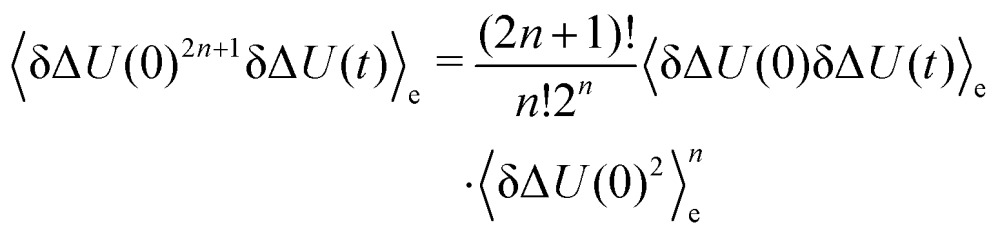
The Stokes shift relaxation function is thus (again) described by eqn (4), but on different mathematical grounds. The absolute Stokes shift ΔΔ*U*, which is Δ*U*(0) – Δ*U*(∞) is then6
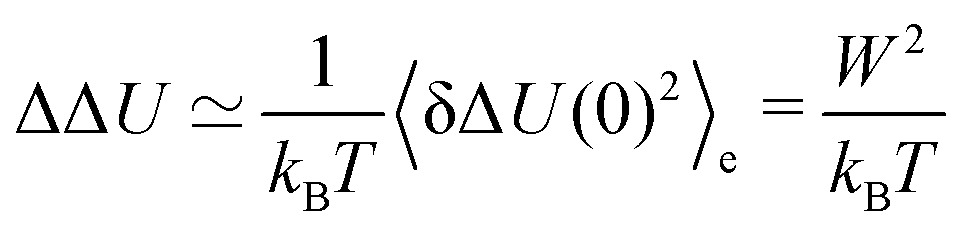
in the linear response approximation, with the spectral width7
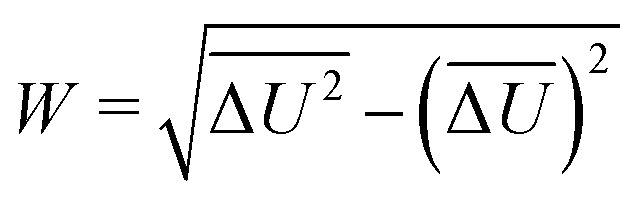



## 3 Methods

Equilibrium simulations in the ground and excited state, as well as nonequilibrium simulations of the solutes coumarin 153 (C153) and *N*-methyl-6-oxyquinolinium betaine (MQ) were carried out in the polar solvents acetonitrile (ACN), methanol (MeOH) and 2-propanol (2-PrOH), as well as in the ionic liquids 1-ethyl-3-methylimidazolium dicyanamide ([Im_21_][DCA]) and 1-ethyl-3-methylimidazolium trifluoromethanesulfonate ([Im_21_][OTf]). The solvents were chosen to cover a wide range of viscosities, as the timescale of the time-dependent Stokes shift, and therefore its range, depends directly on solvent viscosity.^25^ All simulations were conducted with the program package CHARMM.^52^ The force field for MQ was taken from ref. 53. The force field for C153 was set up accordingly, namely the geometry was optimized at the B3LYP 6-311G++(2d,2p) level of theory using density functional theory (DFT) and the respective values put into the intramolecular potentials obtained from PARAMCHEM^54^,^55^ and the CHARMM General Force Field (CGenFF).^56^ The partial charges were calculated using TD-DFT with the ωB97xD hybrid DFT functional^57^ and the CHelpG method.^58^

The force fields of the ionic liquids were taken from Padua and coworkers,^59^–^61^ and made polarizable with atomic polarizabilities from ref. 62. Polarizability is especially important to correctly depict dynamic properties of ionic liquids. For MeOH and 2-PrOH, polarizable force fields were taken from ref. 63 and the CGenFF. For ACN, a suitable nonpolarizable force field was taken from the CGenFF. We note that for polar liquids, polarizable force fields are not always superior to well tuned nonpolarizable forcefields (see also ref. 53), so that this choice should not affect the outcome of this study. All trajectories were calculated using the Velocity-Verlet integrator with a timestep of 1 fs (0.5 fs during *NPT* simulation) and a Nosé–Hoover thermostat.^64^,^65^ Periodic boundary conditions were used, where electrostatic interactions were calculated using the particle mesh Ewald method (grid size 1 Å, cubic splines of order 6, a *κ* of 0.41 Å^–1^) and van der Waals interactions were cut off at 12 Å.

All systems were randomly packed to cubic simulation boxes using PACKMOL^66^ and converged during a subsequent 0.5 ns *NPT* equilibration to the boxlengths listed in Table 1. The equilibrium simulations were conducted at 300 K after an equilibration of 1 to 5 ns for the time periods listed in Table 1. Nonequilibrium simulations were calculated using 500 independent starting configurations obtained from either long *NVT* simulations or independently packed boxes. Where necessary, the starting configurations were further equilibrated for 0.5 ns for the polar solvents, and 1 ns for the ionic liquids per replica. The trajectories were then monitored after sudden change of the partial charge distribution from ground to excited state for time periods according to Table 1. Thus, a total of 4.2 μs of fully polarizable, atomic molecular dynamics simulation was produced in this study, not counting in another few microseconds of equilibration of starting configurations. To the best of our knowledge, no simulation study of the Stokes shift exists on such a large scale. The resulting trajectories were analyzed *via* a python program based on MDAnalysis.^67^ 95% confidence intervals are given, where appropriate, as 
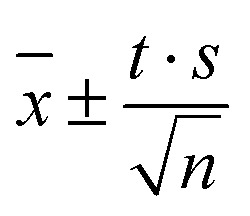
 where *x* is the average of the property of interest, *t* the Student *t* factor, *s* the standard deviation and *n* the number of independent trajectories. For the nonequilibrium simulations, *n* is simply the number of simulated trajectories; for the equilibrium simulation the long trajectory was cut into 5 parts and analyzed separately to yield an upper bound of the true confidence interval.

**Table 1:** Boxlengths of cubic simulation boxes and lengths of simulation of equilibrium (EQ) and nonequilibrium (NEQ) trajectories

Content	Boxlength (Å)	EQ (ns)	NEQ
1 MQ + 1000 ACN	44.40	20	500 × 100 ps
1 C153 + 1000 ACN	44.40	20	500 × 100 ps
1 MQ + 1000 MeoH	40.45	20	500 × 100 ps
1 C153 + 1000 MeOH	40.47	20	500 × 100 ps
1 MQ + 1000 2-ProH	50.61	20	500 × 100 ps
1 C153 + 1000 2-PrOH	50.61	20	500 × 100 ps
1 MQ + 500 [Im_21_][DCA]	51.17	50	500 × 1 ns
1 C153 + 500 [Im_21_][DCA]	51.15	50	500 × 1 ns
1 MQ + 500 [Im_21_][OTf]	53.52	100	500 × 2.5 ns
1 C153 + 500 [Im_21_][OTf]	53.45	100	500 × 2.5 ns

## 4 Results and discussion

### 4.1 Comparison of equilibrium and nonequilibrium relaxation functions and absolute shifts

**Fig. 1:**
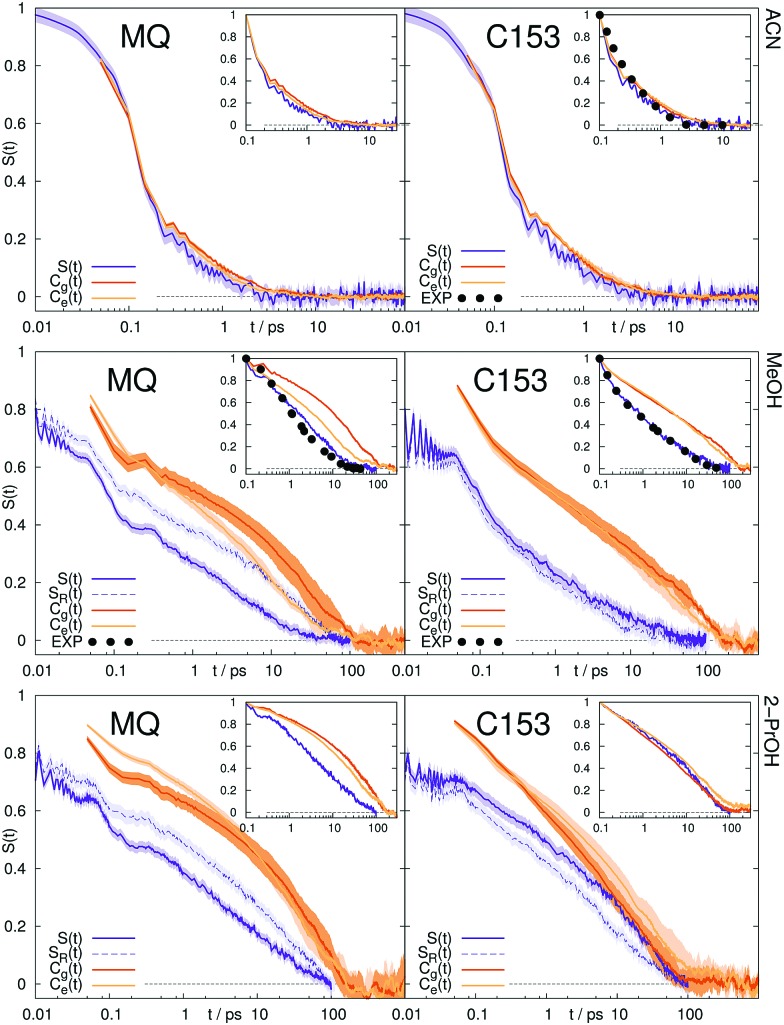
Stokes shift relaxation function after excitation, *S*(*t*), after deexcitation, *S*_R_(*t*), and time correlation functions *C*_g_(*t*) and *C*_e_(*t*) in ground and excited state in acetonitrile (top), methanol (middle) and 2-propanol (bottom) for MQ (left) and C153 (right). The colored area corresponds to a 95% confidence interval. Experimental data (labeled EXP) are taken from ref. 14 and 68.

**Fig. 2:**
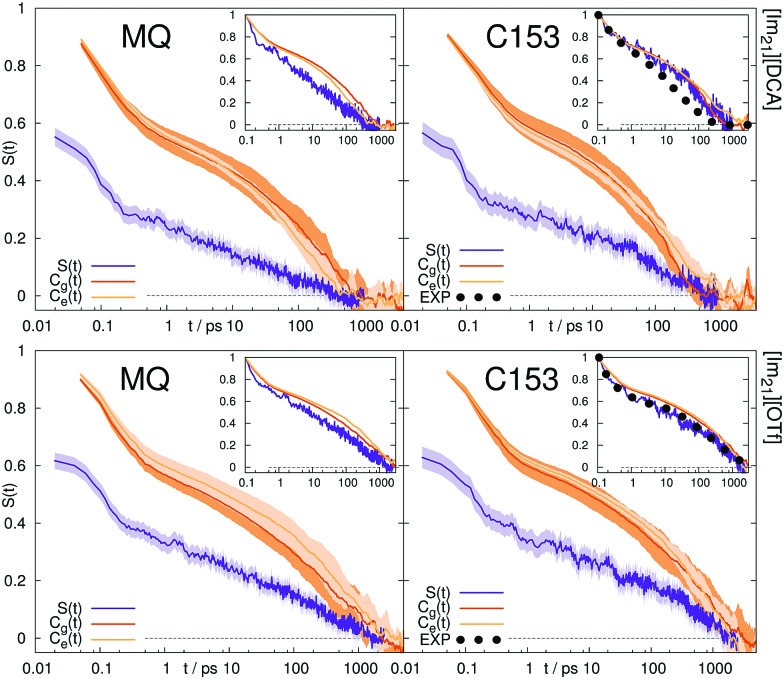
Stokes shift relaxation function *S*(*t*) and time correlation functions *C*_g_(*t*) and *C*_e_(*t*) in ground and excited state in [Im_21_][DCA] (top) and [Im_21_][OTf] (bottom) for MQ (left) and C153 (right). The colored area corresponds to a 95% confidence interval. Experimental data (labeled EXP) are taken from ref. 69.

The Stokes shift relaxation function *S*(*t*) from non-equilibrium simulations, as well as the respective time correlation functions *C*_g_(*t*) and *C*_e_(*t*) from equilibrium simulations are shown in Fig. 1 for polar solvents and in Fig. 2 for ionic solvents. A linear-linear plot (instead of the logarithmic scale of the time axis in Fig. 1) for polar solvents, as well as a plot of the logarithm of the Stokes shift for all solvents can be found in the ESI.†

If conventional linear response theory is applicable, *C*_g_(*t*), *C*_e_(*t*) and *S*(*t*) should correspond well to each other. This is only true for ACN as solvent. For all other solvents and for both chromophores, the full relaxation curves from equilibrium and nonequilibrium simulations do not match. Especially in the case of ionic liquids LRT underestimates the amount of early relaxation. The insets in Fig. 1 and 2 show the relaxation after 0.1 ps, where the curves were set to 1 at 0.1 ps, as experimental data shown here is mostly normalized at this point in time, too. For MQ, this normalization does not lead to a good agreement of *C*_g_(*t*) or *C*_e_(*t*) with the true *S*(*t*) curves, neither in MeOH or 2-PrOH, nor in the ionic liquids [Im_21_][DCA] or [Im_21_][OTf], although in the latter the discrepancy is very small. For the chromophore C153 a different picture arises: The linear response approximation seems to hold after 0.1 ps for the solvents 2-PrOH, [Im_21_][DCA] and [Im_21_][OTf] and only fails in MeOH, for which a failure of linear response is already known.^70^–^72^ An early study of C153 in ACN and MeOH finds contradictory results, namely that linear response theory holds.^73^ However, due to the limited computer power back then, the equilibrium simulations are not long enough to yield statistically significant results for MeOH, so that we attribute our different finding to better sampling. Incidentally, the different solvent force fields contribute to the dissimilar conclusions drawn, too. The effect of sampling and force field in our study, as well as in the system of ref. 73 is given in the ESI.† For C153 in 2-PrOH, [Im_21_][DCA] and [Im_21_][OTf] the curvature at later times is depicted correctly, although the overall response does not depict *S*(*t*). Thus, LRT seems to be applicable for most simulations of C153 for the non-inertial solvation response. The inertial solvation response, however, cannot be described *via* LRT for both chromophores in all studied solvents but ACN. The insets in Fig. 1 and 2 furthermore show the experimentally obtained Stokes shift relaxation functions,^14^,^68^,^69^ which correspond very well to the calculated *S*(*t*) throughout all systems.

**Fig. 3:**
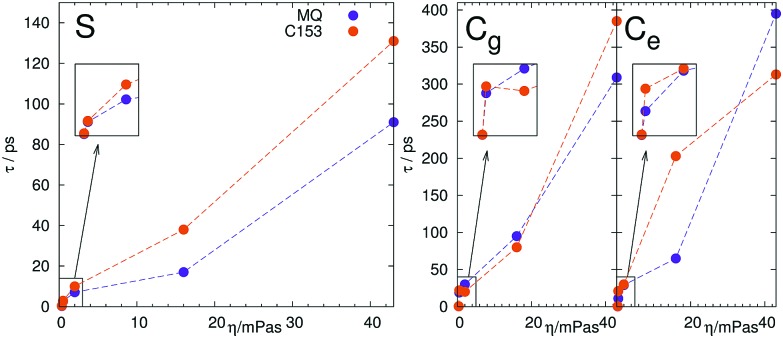
Scaling of the relaxation time *τ* from nonequilibrium (*S*(*t*), left) and equilibrium simulations in the ground (*C*_g_(*t*), middle) and excited state (*C*_e_(*t*), right) with solvent viscosity *η*_exp_ (see Table 2).

To compare the different timescales of solvation, it is convenient to calculate the integral relaxation time8
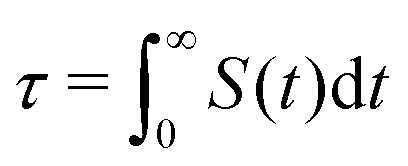
(analogous definition for *C*_g_(*t*) and *C*_e_(*t*)). The relaxation times are listed in Table 2. The relaxation times obtained from nonequilibrium simulations follow the expected trends, namely show an increase of relaxation time with an increase in viscosity of the solvent. Furthermore, the relaxation times of MQ are always less than for the larger chromophore C153, which is expected from the correlation of solute rotation (and thus its volume) and relaxation time in slowly rotating solvents. To visualize this behavior, we plotted the relaxation time against the solvent viscosity, shown in Fig. 3. As mentioned, the relaxation time increases with the viscosity *η*_exp_, MQ relaxes faster than C153 and the difference between them becomes larger as the correlation of solute and solvent rotation increases. The relaxation times obtained from equilibrium simulations, however, raise a number of problems. First, they overestimate the timescale of solvation by a factor of two to eight. Second, they do not scale correctly with viscosity, for example C153 in 2-PrOH relaxes faster than in MeOH. Third, MQ relaxes not always faster than C153, so that the lines in Fig. 3 sometimes decrease or intersect each other. Thus, the absolute timescales provided by equilibrium simulations are not an accurate reflection of reality.

**Table 2:** Relaxation times *τ* of the Stokes shift relaxation function from nonequilibrium (*S*(*t*)) and equilibrium simulations in the ground (*C*_g_(*t*)) and excited state (*C*_e_(*t*)). Experimental viscosities^74^–^77^ at 298.15 K

	*η*_exp_ [mPa s]	*τ*_MQ_ [ps]			*τ*_C153_ [ps]		
		*S*	*C*_g_	*C*_e_	*S*	*C*_g_	*C*_e_
Overall response, normalized at 0 ps							
ACN	0.35	0.32	0.43	0.36	0.47	0.50	0.53
MeOH	0.53	2.7	19	11	2.9	22	21
2-PrOH	2.05	7.1	30	29	10	20	30
[Im_21_][DCA]	16.1	17	95	65	38	80	203
[Im_21_][OTf]	42.9	91	309	395	131	398	313

Response in the diffusive regime, normalized at 0.1 ps							
ACN	0.35	0.33	0.62	0.48	0.47	0.67	0.79
MeOH	0.53	5.8	29	16	5.8	30	28
2-PrOH	2.05	13	40	34	15	15	26
[Im_21_][DCA]	16.1	44	124	82	97	97	199
[Im_21_][OTf]	42.9	178	352	465	252	429	362

Since we pointed out earlier that the inertial relaxation is not described correctly *via* LRT in all solvents but ACN, timescales of the relaxation after 0.1 ps are also given in Table 2. They are calculated as the integral of *S*(*t*) normalized at 0.1 ps (*i.e.* the area under the curve depicted in the insets in Fig. 1 and 2). The obtained relaxation times describe the timescale of diffusive contributions and collective rotation.^78^,^79^ Yet again the same problems arise, namely the use of LRT falsely leads to the assumption that the excited state C153 is solvated faster in 2-PrOH than in MeOH. Similarly, LRT claims that MQ in 2-PrOH or [Im_21_][DCA] is solvated on a slower timescale than C153, which is not observed from nonequilibrium simulations.

Apart from the timescales discussed above, also the absolute Stokes shift ΔΔ*U* obtained from equilibrium and non-equilibrium simulations should be identical if a system exhibits a linear solvation response. Table 3 lists the absolute shifts obtained from non-equilibrium and equilibrium simulations, as well as experimental absolute shifts from ref. 14 and 69. The shifts ΔΔ*U* were calculated as *h*[*ν*(0) – *ν*(∞)] from experiment, as Δ*U*(0) – Δ*U*(∞) from nonequilibrium simulations and *via*eqn (6) from equilibrium simulations. For the polar solvents, LRT reproduces the absolute shifts from nonequilibrium simulations and experiment, even where the curvature of the LRT Stokes shift relaxation function is quantitatively wrong (*e.g.* for 1MQ or C153 in MeOH). For the ionic liquids, the linear response approximation overestimates the absolute shifts by a factor of 2 to 3.

**Table 3:** Absolute Stokes shift ΔΔ*U* in kJ mol^–1^ obtained from non-equilibrium simulations (*S*(*t*)) and equilibrium simulations in the ground (*C*_g_(*t*)) and excited (*C*_e_(*t*)) state, as well as experimental Stokes shift *via h*[*ν*(∞) – *ν*(0)] from ref. 14 and 69

System		ΔΔ*U*_*C*_g__	ΔΔ*U*_*C*_e__	ΔΔ*U*_S_	ΔΔ*U*_exp_
MQ	ACN	27	30	27	
	MeOH	52	48	52	42
	2-PrOH	44	46	44	
	[Im_21_][DCA]	77	79	38	
	[Im_21_][OTf]	67	72	38	

C153	ACN	32	30	27	27
	MeOH	40	39	35	37
	2-PrOH	21	25	28	
	[Im_21_][DCA]	101	96	36	25
	[Im_21_][OTf]	81	83	33	28

**Fig. 4:**
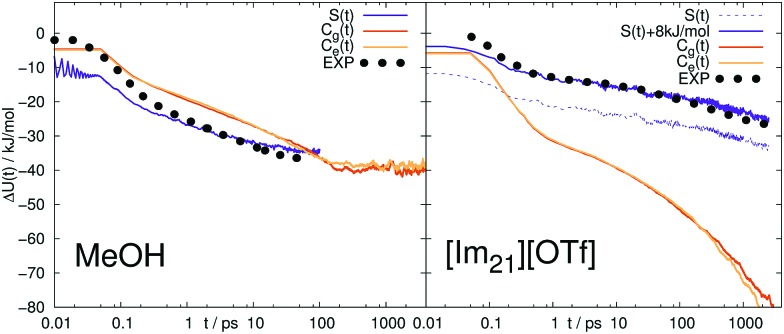
Absolute change in energy Δ*U*(*t*) calculated from the Stokes shift relaxation function *S*(*t*) and time correlation functions *C*_g_(*t*) and *C*_e_(*t*) in ground and excited state in MeOH (left) and [Im_21_][OTf] (right) for C153. Experimental data (labeled EXP) are taken from ref. 14 and 69.

Thus, the validity of LRT even for coumarin 153 in ionic liquids is not confirmed, in contrast to the general findings in ref. 32. The implications of the overestimation of the overall shift by the time correlation functions become also visible when the Stokes shift relaxation function is plotted unnormalized, as shown in Fig. 4 for C153 in MeOH and [Im_21_][OTf]. In MeOH, LRT correctly reproduces the absolute shift, so that both the normalized and unnormalized relaxation functions show the same trend. Again, the nonequilibrium simulations fit very well to experimental data, which is remarkable given the fact that the curves are not normalized and were not set to the same value at a specific point. In [Im_21_][OTf], the relaxation functions obtained *via* LRT does not describe neither the nonequilibrium counterpart nor experiment. Note that *S*(*t*) is plotted twice, once set to 0 at 0 fs, and once at 14 fs, which results in a constant offset of about 8 kJ mol^–1^. As the experimental resolution is about 80 fs, and the value at time 0 is only estimated, both representations of *S*(*t*) are equally valid and produce a very good fit to experiment.

**Fig. 5:**
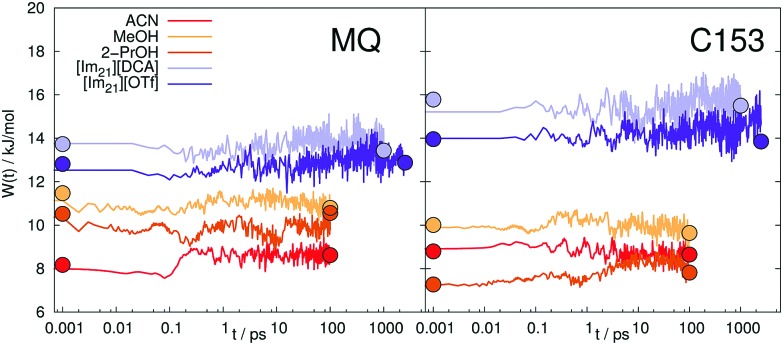
Time-evolution of the spectral width *W*(*t*) of the interaction energy between MQ (left) or C153 (right) and different solvents. The circles at the edges of the curves represent the corresponding widths of the equilibrium simulations.

The magnitude of the absolute Stokes shift is directly connected to the width of the energy gap distribution within linear response theory^80^,^81^ *via*eqn (6). Different absolute shifts ΔΔ*U* therefore correspond to different widths of the distribution of Δ*U*. As obvious from Table 3, the studied systems do not show large changes of the width. Within the linear response framework, the width of the energy gap in the ground and excited state cannot differ, so that a change in width is directly connected to a nonlinear response.^81^,^82^ In ref. 36 we observed furthermore that also large intermediate changes in *W*(*t*), a so called intermediate broadening of the spectral width can cause deviations from LRT. Fig. 5 shows the time-evolution of the spectral width obtained from nonequilibrium simulations, as well as the widths in equilibrium.

In contrast to small artificial solutes, which are known to show non-stationary statistics (intermediate broadening),^4^,^36^,^83^ which correlates with LRT failure, the real chromophores MQ and C153 do not show significant intermediate broadening. There is also no large difference between the spectral widths in the equilibrated states (circles in Fig. 5). The observed failure of LRT can therefore not be attributed to time-dependent changes of the energy gap distribution and has to be sought elsewhere. We furthermore note that the evaluation of the width might be biased, since the sampling of some configurations *via* a long MD run is statistically hampered, similar to observations of King and Warshel.^84^ For the large chromophores MQ and C153 undergoing only minor changes upon excitation such an effect should be small, but might be non-negligible for smaller artificial solutes, for example in ref. 4 and 36.

To sum up, from the ten studied systems only MQ and C153 in ACN showed to completely obey LRT, although the interaction energy between solute and solvent is locally Gaussian and stationary in all systems. LRT mainly fails to describe the inertial solvation response, but also leads to deviations on the timescales of diffusion and rotation. We note that C153 in 2-PrOH shows only (comparably) small deviations. To further examine the validity of LRT in the polar solvents, we calculated the nonequilibrium response of the deexcitation of the chromophore in MeOH and 2-PrOH. In the case of linear response, the timescales of solvent relaxation after excitation and deexcitation of a solute should be indistinguishable. The respective relaxation functions are shown in Fig. 1 as dashed curve, labeled *S*_R_. For both chromophores, the forward and backward reaction produce different timescale of rearrangement. A lowering of the dipole moment produces for all four solute–solvent combinations a faster solvent rearrangement than a strengthening. In other words, a decrease in order is faster accomplished than an increase in order. The discrepancy between *S*(*t*) and *S*_R_(*t*) is large for MQ, but very small for C153. A closer look at the forward and reverse excitation of C153 in MeOH reveals another interesting fact: Although *C*_g_(*t*) = *C*_e_(*t*), and *S*(*t*) ≃ *S*_R_(*t*), which is typical for a linear response,^3^,^85^ the time correlation functions from equilibrium simulations are not capable of describing the correct timescale or curvature of *S*(*t*). This effect was also found by ref. 7, and we therefore note that neither *C*_g_(*t*) = *C*_e_(*t*) nor *S*(*t*) ≃ *S*_R_(*t*) can give insights on the validity of LRT.

### 4.2 Partial correlation functions

**Fig. 6:**
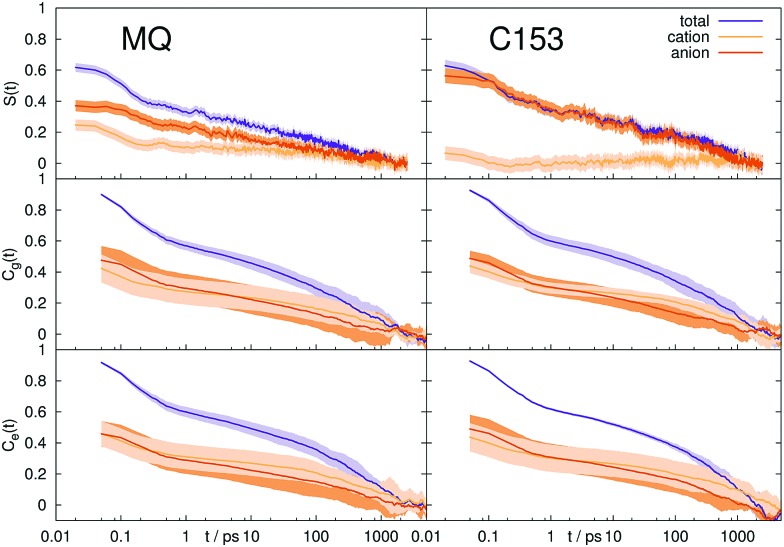
Contributions from cations and anions to the Stokes shift relaxation function *S*(*t*) (top panel) and time correlation functions *C*_g_(*t*) (middle panel) and *C*_e_(*t*) (bottom panel) in ground and excited state in [Im_21_][OTf] for MQ (left) and C153 (right). The colored area corresponds to a 95% confidence interval.

To disentangle the contributions from anions and cations to the overall solvation response, the relaxation function *S*(*t*) can easily be separated, as Δ*U* is simply a sum of pair interactions, so that9
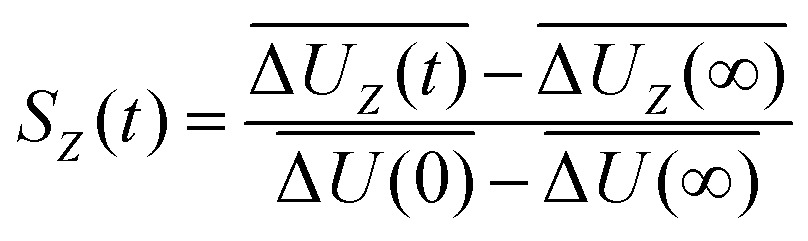
where *Z* can be any contribution, here from cation movement and anion movement. The contributions in equilibrium simulations can be obtained *via* the partial correlation function10
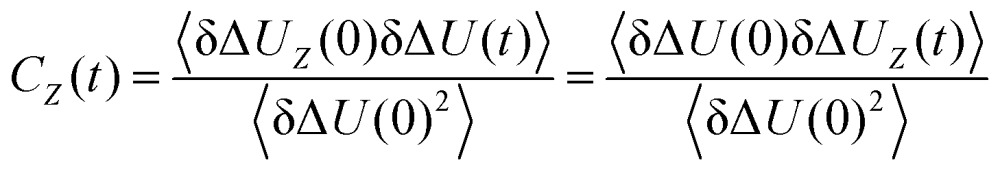
which directly corresponds to *S*_*Z*_(*t*). Autocorrelation functions of the different contributions are not comparable to *S*_*Z*_(*t*), since such a partitioning leads to large cross-correlations. Fig. 6 shows the contributions from cations and anions obtained from nonequilibrium and equilibrium simulations in [Im_21_][OTf]. The corresponding figure for [Im_21_][DCA] can be found in the ESI.† For MQ in the nonequilibrium simulations the anions contribute more to the overall response and their movement occurs on a longer timescale, so that the anionic contribution rises with time. The equilibrium simulations, however, do not depict this behavior. Here, the response is made up one half by anion movement, the other half by cation movement which occurs on the same timescale, and the respective contributions do not change in time. For C153, this discrepancy becomes even larger, as the cationic contribution falls to 0 within 1 ps in the nonequilibrium simulation, but contributes nearly 50% to the overall Stokes shift on all timescales in the equilibrium simulation. Here, LRT is clearly not capable of depicting accurate magnitudes of cationic and anionic contributions. A similar phenomenon (a hidden breakdown of linear response), where *S*(*t*) and *C*(*t*) correspond approximately to each other, but individual contributions do not obey linear response, was already observed by Schwartz and coworkers for rotational and translational contributions to the solvation response.^86^,^87^

### 4.3 Higher-order correlation functions

**Fig. 7:**
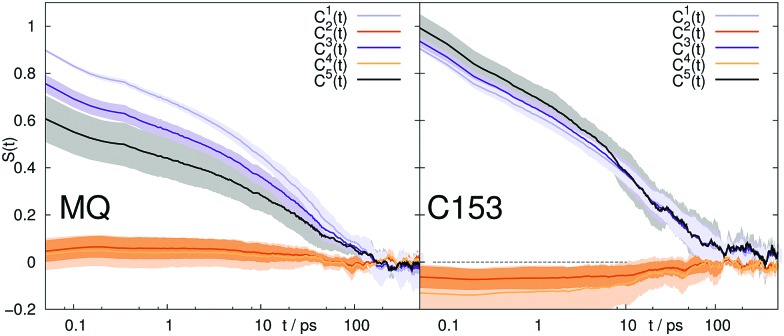
Higher order correlation functions *C*^*n*^, where 
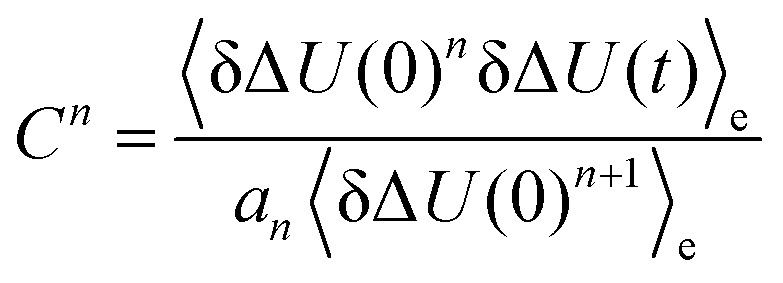
 and *a*_1_ = 1, *a*_2_ = 1.6, *a*_3_ = 3, *a*_4_ = 6.4 and *a*_5_ = 15 according to eqn (5) after rearrangement. System: 2-PrOH for MQ (left) and C153 (right). The colored area corresponds to a 95% confidence interval.

Sometimes the applicability of LRT is tested *via* the validity of eqn (5).^35^,^50^,^88^ We thus calculated higher-order correlation functions for all systems up to the order of five, both for the overall response, as well as for the cationic and anionic contributions. The corresponding data is shown in Fig. 7 for 2-PrOH and in the Supporting Information^†^ for all other solvents. Despite deviations from eqn (5) for MQ in 2-PrOH in the odd order correlation functions, and for C153 in 2-PrOH in the even order correlation functions, the relation seems to hold for all other systems within the 95% confidence interval given.

Thus, this analysis of higher-order correlations points towards applicability of LRT for all solvent but 2-PrOH. As we have seen earlier, LRT fails (*i.e. S*(*t*) does not equal *C*(*t*)) for all systems but ACN, so that observing higher-order correlation functions in accordance to eqn (5) does not guarantee validity of the linear response formalism, and should thus be treated very cautiously. Since only the first few higher-order correlations can be calculated with acceptable signal-to-noise ratio and reasonable confidence intervals, we miss any deviations from eqn (5) in all correlation functions of order higher than 5 (odd) or higher than 4 (even). Furthermore, if the equilibrium trajectory does not sample all important configurations,^84^*i.e.* ergodicity is not reached, *C*(*t*) might not resemble *S*(*t*) even if eqn (5) is obeyed.

**Fig. 8:**
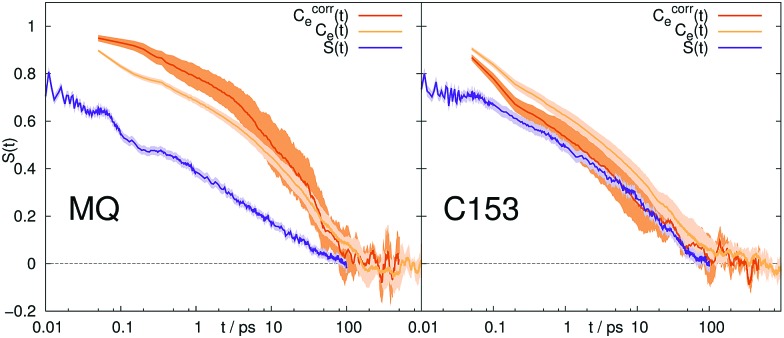
Normalized correction *C*corre to the time correlation function *C*_e_(*t*) according to eqn (11) in 2-PrOH for MQ (left) and C153 (right). The colored area corresponds to a 95% confidence interval.

Recently, corrections to the correlation function *via* higher-order correlations were suggested for systems were eqn (5) is not fully obeyed. We therefore calculated corrections up to the order of five, *via* the suggested scheme from ref. 88,11
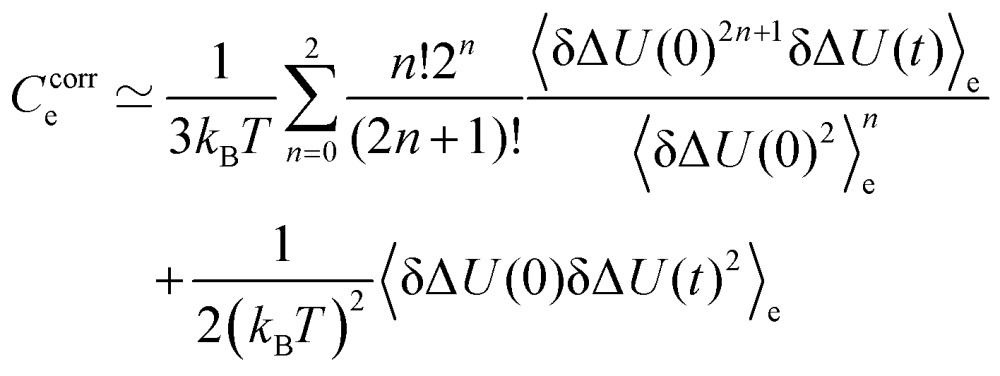
where the first term can be directly derived from eqn (5), and is a simple average of the first three uneven correlation functions. The first even term (second order) is included *via* the second term. The inclusion of higher-order correlation functions of order 4 was omitted, as the signal-to-noise ratio was unacceptably low. To normalize this function, it is simply divided by its value at *t* = 0. Fig. 8 shows the uncorrected and corrected correlation functions, as well as the nonequilibrium results for both chromophores in 2-PrOH.

For C153 the corrected correlation function comes closer to the true nonequilibrium results, yielding nearly quantitative agreement. This improvement is reasonable, since the even order higher-correlation functions are nonzero and contribute to the observed solvation response. Thus, solvation dynamics of C153 in 2-PrOH can be roughly described within the corrected linear response formalism. However, for all other systems (shown in the ESI†), the correction does not provide a significant improvement and sometimes even moves the curve in the wrong direction.

Furthermore we calculated higher-order correlation functions for the cation and anion contributions to the overall response (data shown in the ESI†). Again, eqn (5) is obeyed within the confidence interval, so that the previously observed failure of LRT for the partial correlation functions could not have been predicted by the partial higher-order correlation functions.

### 4.4 Influence of solvent structure

**Fig. 9:**
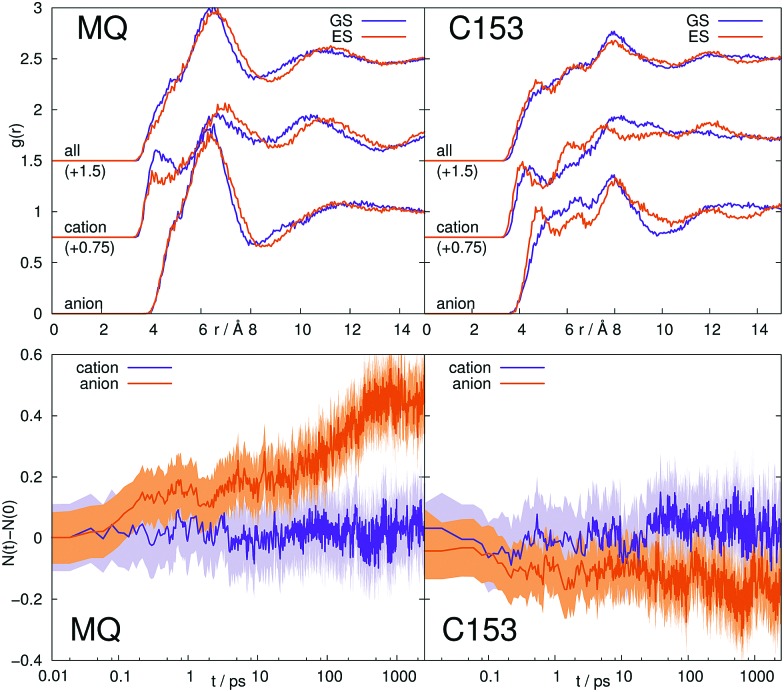
Top: Radial distribution functions of solvent molecules around the central solute MQ (left), or C153 (right) in [Im_21_][OTf]. Bottom: Change in number of first shell ions around the central solute for the same system. The colored area corresponds to a 95% confidence interval.

Whenever the structure of the solvent changes significantly after excitation of the solute, LRT is likely to fail.^70^–^72^,^89^,^90^ We therefore calculated the radial distribution functions *g*(*r*) around MQ and C153 in the ground and excited state in all five solvents, as well as the time evolution of the cation and anion coordination number *via* Voronoi tessellation. Fig. 9 shows the corresponding results for both chromophores in [Im_21_][OTf], all other solvents are shown in the ESI.†

The excitation of the solute causes slight adaption of the solvent structure in all systems, where MQ inflicts larger structure changes in the surrounding solvent than C153. In polar solvents, this trend can be seen directly from the radial distribution functions, and in ionic liquids from the change in coordination numbers after excitation. For example, after excitation of MQ in [Im_21_][OTf] (left panels in Fig. 9), the anion coordination number increases by approximately 0.5, with only moderate changes in the structure of the closest molecules seen in *g*(*r*). The excitation of C153 in ionic liquids, in contrast, induces larger structure changes of the few innermost ions (change of peak positions and intensities in the radial distribution functions), but the coordination number does not change significantly. Note that the first solvation shell extends to quite large distances, here up to about 8 Å. For MQ in MeOH, 2-PrOH, [Im_21_][DCA] and [Im_21_][OTf], as well as C153 in [Im_21_][DCA] and [Im_21_][OTf] a failure of LRT could be due to rearrangements in the solvent structure visible in the radial distribution functions or ion coordination number. C153 in polar solvents, however, does not produce significant changes in structure, so that for these system, validity of LRT is expected, at least from this point of view. The observed failure of the linear response approximation of C153 in MeOH is thus not predictable from a structure analysis *via* radial distribution functions.

We furthermore calculated the number of hydrogen bonds in the equilibrated ground and excited state, as well as the time dependency of the number of hydrogen bonds after excitation of the solute (in nonequilibrium), Table 4. Criterias for hydrogen bonds (maximum distance of 2.2 Å, minimum angle of 130 Å) were taken from Hunt *et al.*^91^ Possible hydrogen bond donors are the hydroxyl hydrogens in MeOH and 2-PrOH, as well as the three hydrogens attached to the imidazolium ring in [Im_21_][DCA] and [Im_21_][OTf]. Possible acceptors are the oxygen atoms in MQ and C153 (no hydrogen bonds were found for the nitrogen atom in C153).

**Table 4:** Number of hydrogen bonds in the equilibrated ground and excited state, as well as timescale of the relaxation to the new number of hydrogen bonds after excitation (or deexcitation) of the solute, *τ*_relax_, where applicable

	# of H-bonds		*τ*_relax_ [ps]	
	GS	ES	GS → ES	ES → GS
MQ				
MeOH	2.48 ± 0.07	1.19 ± 0.06	5.9	19.7
2-PrOH	2.72 ± 0.05	1.58 ± 0.08	12.2	21.5
[Im_21_][DCA]	0.017 ± 0.002	0.005 ± 0.001	—	—
[Im_21_][OTf]	0.016 ± 0.006	0.008 ± 0.002	—	—

C153				
MeOH	0.77 ± 0.03	1.36 ± 0.06	12.3	7.8
2-PrOH	1.10 ± 0.04	1.83 ± 0.04	19.5	12.2
[Im_21_][DCA]	0.002 ± 0.001	0.003 ± 0.001	—	—
[Im_21_][OTf]	0.002 ± 0.001	0.003 ± 0.001	—	—

**Fig. 10:**
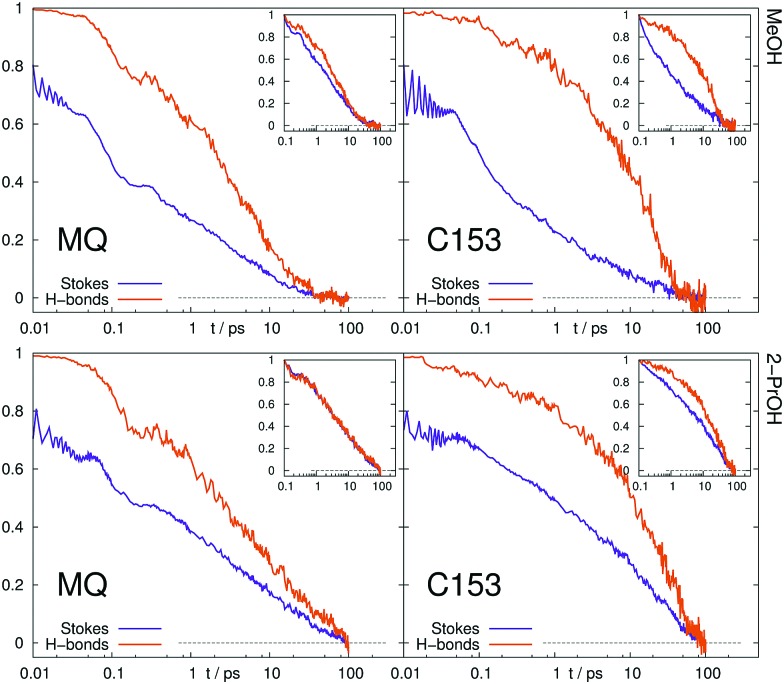
Time evolution of normalized number of hydrogen bonds compared to the normalized Stokes shift. Inset: Normalization at 0.1 ps.

In ionic liquids, hydrogen bonds occur neither in the ground, nor in the excited state of MQ and C153. In MeOH and 2-PrOH, the number of hydrogen bonds varies between the equilibrated ground and excited state. For example, MQ has on average 2.5 hydrogen bonds in the ground state and eventually loses 1.3 hydrogen bonds after excitation. C153, in contrast increases the number of hydrogen bonds after excitation. The timescale of the decrease or increase in hydrogen bonds after excitation is also given in Table 4. Throughout all systems, a decrease of the number of hydrogen bonds is faster than an increase. The normalized number of hydrogen bonds after excitation is shown in Fig. 10, where the number of hydrogen bonds in the equilibrated ground state (instance of excitation) corresponds to 1, and in the equilibrated excited state to 0. For comparison, the timescale of solvent relaxation *via* the time-dependent Stokes shift is shown, too. Disregarding the inertial part of the response (before 0.1 ps) the rearrangement of hydrogen bonds occurs approximately on the same timescale as the overall solvent relaxation for MQ. In contrast, the hydrogen bonds after excitation of C153 rearrange slower than the overall solvent relaxation.

A change in number of hydrogen bonds and the corresponding timescale cannot be predicted by equilibrium simulations, so that linear response theory is likely to fail for MQ and C153 in MeOH and 2-PrOH. A nonlinear response associated with different hydrogen bonding patterns in the ground and excited was also previously observed by Ladanyi and coworkers for artificial diatomic solutes in methanol.^70^–^72^ The observed failure of LRT for C153 in MeOH and 2-PrOH is thus plausible, as the hydrogen bonding structure changes after excitation.

## 5 Conclusion

We used simulations of two well known fluorescence probes, MQ and C153, in the polar liquids ACN, MeOH and 2-PrOH, as well as in the ionic liquids [Im_21_][DCA] and [Im_21_][OTf] to assess the validity of linear response approximations for different solute–solvent combinations. The solvation response from nonequilibrium simulations perfectly reproduced experimental data for all systems (where experimental data exists), even on an absolute scale. Comparison of equilibrium and nonequilibrium solvation response functions showed that LRT neither yields the correct timescale nor absolute magnitude of the time-dependent Stokes shift for most systems, where LRT fails most severely for the inertial solvation response. A correction to the linear response estimate proposed by Thompson and coworkers^88^ proved to be helpful only in one of all systems studied (C153 in 2-PrOH). Furthermore, the cationic and anionic contributions to the overall shift in the case of ionic liquids could not be reproduced with LRT, yielding quantitatively wrong results.

One should therefore ask whether this failure of LRT could have been predicted from equilibrium simulations. In literature, often the computation of higher-order correlation functions and their factorizations is proposed as a test for LRT validity.^35^,^50^,^88^ We found, however, that most of the system studied here easily pass this test, although a comparison of equilibrium and nonequilibrium response curves reveals a severe failure of LRT. Likewise, the magnitude of corrections to the time-correlation function *via* higher-order correlation function cannot be used to predict LRT failure or applicability. Also the existence of a local Gaussian distribution of the energy gap in the ground and excited state, and even its stationarity did not guarantee *C*(*t*) ≃ *S*(*t*) in all cases studied, *i.e.* did not prove a global gaussian energy gap. We also found systems, where *C*_e_(*t*) ≃ *C*_g_(*t*) and *S*(*t*) ≃ *S*_R_(*t*), that is, a forward and reverse change in solute electron distribution leads to the same response, but *C*(*t*) ≠ *S*(*t*). A good estimate of LRT applicability was found to be the solvent structure in the ground and excited state, *i.e.* the radial distribution function, coordination number and number of hydrogen bonds. Whenever a large change in solvent structure occurs after excitation, LRT is likely to fail.

For a clear assessment of the validity of LRT in a system the absolute and normalized LRT response function should be compared to experiment or nonequilibrium simulation results. Whenever no experimental data is available for a system, we recommend to calculate the solvent structure in the ground and excited state. If no large changes are found, the time evolution of the spectral width, as well as the absolute solvation response should be calculated from a small number of nonequilibrium simulations and compared to the LRT prediction. Although a small ensemble of nonequilibrium simulations might not yield statistically significant results itself, the magnitude of the shift, the approximate curvature of the response function and changes in the spectral width can easily be compared to the respective results from equilibrium simulations and give a reliable impression of the applicability of LRT. The use of linear response approximations to describe the solvation dynamics of real chromophores in real solvents without a careful testing of its applicability should not be encouraged. We also note that solvation dynamics around real chromophores is a lot more difficult to predict than around simple artificial solutes found in literature.^4^,^35^,^36^,^70^,^88^

## Conflicts of interest

There are no conflicts to declare.

## Supplementary Material

Supplementary information

## References

[cit1] Bagchi B., Oxtoby D. W., Fleming G. R. (1983). Chem. Phys..

[cit2] Maroncelli M., Fleming G. R. (1987). J. Chem. Phys..

[cit3] Maroncelli M. (1991). J. Chem. Phys..

[cit4] Carter E. A., Hynes J. T. (1991). J. Chem. Phys..

[cit5] Maroncelli M. (1993). J. Mol. Liq..

[cit6] Chapman C. F., Fee R. S., Maroncelli M. (1995). J. Phys. Chem..

[cit7] Skaf M. S., Ladanyi B. M. (1996). J. Phys. Chem..

[cit8] Mertz E. L., Tikhomirov V. A., Krishtalik L. I. (1997). J. Phys. Chem. A.

[cit9] Sonoda M. T., Moneira N. H., Martínez L., Favero F. W., Vechi S. M., Martins L. R., Skaf M. S. (2004). Braz. J. Phys..

[cit10] Lustres J. L. P., Kovalenko S. A., Mosquera M., Senyushkina T., Flasche W., Ernsting N. P. (2005). Angew. Chem., Int. Ed..

[cit11] Ingrosso F., Ladanyi B. M., Mennucci B., Elola M. D., Tomasi J. (2005). J. Phys. Chem. B.

[cit12] Bagchi B., Jana B. (2010). Chem. Soc. Rev..

[cit13] Nome R. A. (2010). J. Braz. Chem. Soc..

[cit14] Sajadi M., Weinberger M., Wagenknecht H.-A., Ernsting N. P. (2011). Phys. Chem. Chem. Phys..

[cit15] Allolio C., Sajadi M., Ernsting N. P., Sebastiani D. (2013). Angew. Chem., Int. Ed..

[cit16] Petrone A., Donati G., Caruso P., Rega N. (2014). J. Am. Chem. Soc..

[cit17] Karmakar R., Samanta A. (2002). J. Phys. Chem. A.

[cit18] Ingram J. A., Moog R. S., Ito N., Biswas R., Maroncelli M. (2003). J. Phys. Chem. B.

[cit19] Sha S., Mandal P. K., Samanta A. (2004). Phys. Chem. Chem. Phys..

[cit20] Mandal P. K., Saha S., Karmakar R., Samanta A. (2006). Curr. Sci..

[cit21] Arzhantsev S., Jin H., Baker G. A., Maroncelli M. (2007). J. Phys. Chem. B.

[cit22] Jin H., Baker G. A., Arzhantsev S., Dong J., Maroncelli M. (2007). J. Phys. Chem. B.

[cit23] Shim Y., Kim H. J. (2008). J. Phys. Chem. B.

[cit24] Samanta A. (2010). J. Phys. Chem. Lett..

[cit25] Roy D., Maroncelli M. (2012). J. Phys. Chem. B.

[cit26] Maroncelli M., Zhang X.-X., Liang M., Roy D., Ernsting N. P. (2012). Faraday Discuss..

[cit27] Schmollngruber M., Schröder C., Steinhauser O. (2013). J. Chem. Phys..

[cit28] Daschakraborty S., Biswas R. (2013). J. Chem. Phys..

[cit29] Shim Y., Kim H. J. (2013). J. Phys. Chem. B.

[cit30] Kobrak M. N. (2006). J. Chem. Phys..

[cit31] Shim Y., Duan J., Choi M. Y., Kim H. J. (2003). J. Chem. Phys..

[cit32] Shim Y., Choi M. Y., Kim H. J. (2005). J. Chem. Phys..

[cit33] Zwanzig R., Nonequilibrium Statistical Mechanics, Oxford Univ. Press, New York, 2001.

[cit34] Laird B. B., Thompson W. H. (2007). J. Chem. Phys..

[cit35] Laird B. B., Thompson W. H. (2011). J. Chem. Phys..

[cit36] Heid E., Moser W., Schröder C. (2017). Phys. Chem. Chem. Phys..

[cit37] Margulis C. J. (2004). Mol. Phys..

[cit38] Bhargava B. L., Balasubramanian S. (2005). J. Chem. Phys..

[cit39] Shim Y., Jeong D., Choi M. Y., Kim H. J. (2006). J. Chem. Phys..

[cit40] Kobrak M. N. (2007). J. Chem. Phys..

[cit41] Shim Y., Kim H. J. (2007). J. Phys. Chem. B.

[cit42] Terranova Z. L., Corcelli S. A. (2013). J. Phys. Chem. B.

[cit43] Wu E. C., Kim H. J. (2016). J. Phys. Chem. B.

[cit44] Hu Z., Margulis C. J. (2006). PNAS.

[cit45] Kashyap H. K., Biswas R. (2010). J. Phys. Chem. B.

[cit46] Pal T., Biswas R. (2014). J. Chem. Phys..

[cit47] Chowdhury P. K., Halder M., Sanders L., Calhoun T., Anderson J. L., Armstrong D. W., Song X., Petrich J. W. (2004). J. Phys. Chem. B.

[cit48] Kobrak M. N., Znamenskiy V. (2004). Chem. Phys. Lett..

[cit49] Arzhantsev S., Jin H., Ito N., Maroncelli M. (2006). Chem. Phys. Lett..

[cit50] Li T., Kumar R. (2015). J. Chem. Phys..

[cit51] Wick G. C. (1950). Phys. Rev..

[cit52] Brooks B. R., Brooks III C. L., MacKerell Jr. A. D., Nilsson L., Petrella R. J., Roux B., Won Y., Archontis G., Bartels C., Boresch S., Caflisch A., Caves L., Cui Q., Dinner A. R., Feig M., Fischer S., Gao J., Hodoscek M., Im W., Kuczera K., Lazaridis T., Ma J., Ovchinnikov V., Paci E., Pastor R. W., Post C. B., Pu J. Z., Schaefer M., Tidor B., Venable R. M., Woodcock H. L., Wu X., Yang W., York D. M., Karplus M. (2009). J. Comput. Chem..

[cit53] Heid E., Harringer S., Schröder C. (2016). J. Chem. Phys..

[cit54] Vanommeslaeghe K., MacKerell Jr. A. D. (2012). J. Chem. Inf. Model..

[cit55] Vanommeslaeghe K., Raman E. P., MacKerell Jr. A. D. (2012). J. Chem. Inf. Model..

[cit56] Vanommeslaeghe K., Hatcher E., Acharya C., Kundu S., Zhong S., Shim J., Darian E., Guvench O., Lopes P., Vorobyov I., MacKerell Jr. A. D. (2010). J. Comput. Chem..

[cit57] Chai J.-D., Head-Gordon M. (2008). Phys. Chem. Chem. Phys..

[cit58] Breneman C. M., Wiberg K. B. (1990). J. Comput. Chem..

[cit59] Canongia Lopes J. N. A., Costa Gomes M. F., Pádua A. A. H. (2006). J. Phys. Chem. B.

[cit60] Canongia Lopes J. N., Deschamps J., Pádua A. A. H. (2004). J. Phys. Chem. B.

[cit61] Canongia Lopes J. N., Pádua A. A. H. (2004). J. Phys. Chem. B.

[cit62] Bernardes C. E. S., Shimizu K., Lopes J. C., Marquetand P., Heid E., Steinhauser O., Schröder C. (2016). Phys. Chem. Chem. Phys..

[cit63] Kaminski G., Jorgensen W. L. (1996). J. Phys. Chem..

[cit64] Nosé S. (1984). J. Chem. Phys..

[cit65] Hoover W. G. (1985). Phys. Rev. A: At., Mol., Opt. Phys..

[cit66] Martínez L., Andrade R., Birgin G., Martínez J. M. (2009). J. Comput. Chem..

[cit67] Michaud-Agrawal N., Denning E. J., Woolf T. B., Beckstein O. (2011). J. Comput. Chem..

[cit68] Horng M. L., Gardecki J. A., Papazyan A., Maroncelli M. (1995). J. Phys. Chem..

[cit69] Zhang X.-X., Liang M., Ernsting N. P., Maroncelli M. (2013). J. Phys. Chem. B.

[cit70] Fonseca T., Ladanyi B. M. (1991). J. Phys. Chem..

[cit71] Phelps D. K., Weaver M. J., Ladanyi B. M. (1993). J. Chem. Phys..

[cit72] Fonseca T., Ladanyi B. M. (1994). J. Mol. Liq..

[cit73] Kumar P. V., Maroncelli M. (1995). J. Chem. Phys..

[cit74] Moumouzias G., Panopoulos D. K., Ritzoulis G. (1991). J. Chem. Eng. Data.

[cit75] Komarenko V. G., Manzheli V. G., Radtsig A. V. (1967). Ukr. Fiz. Zh. (Russ. Ed.).

[cit76] Paez S., Contreras M. (1989). J. Chem. Eng. Data.

[cit77] Freire M. G., Teles A. R. R., Rocha M. A. A., Schroder B., Neves C. M. S. S., Carvalho P. J., Evtuguin D. V., Santos L. M. N. B. F., Coutinho J. A. P. (2011). J. Chem. Eng. Data.

[cit78] Heid E., Schröder C. (2017). J. Phys. Chem. B.

[cit79] Gerecke M., Richter C., Quick M., Ioffe I., Mahrwald R., Kovalenko S. A., Ernsting N. P. (2017). J. Phys. Chem. B.

[cit80] Li T. (2014). J. Phys. Chem. B.

[cit81] Stephens M. D., Saven J. G., Skinner S. L. (1997). J. Chem. Phys..

[cit82] Tachiya M. (1989). J. Phys. Chem..

[cit83] Geissler P. L., Chandler D. (2000). J. Chem. Phys..

[cit84] King G., Warshel A. (1990). J. Chem. Phys.

[cit85] Turi L., Mináry P., Rossky P. J. (2000). Chem. Phys. Lett..

[cit86] Bedard-Hearn M. J., Larsen R. E., Schwartz B. J. (2003). J. Phys. Chem. A.

[cit87] Bedard-Hearn M. J., Larsen R. E., Schwartz B. J. (2003). J. Phys. Chem. B.

[cit88] Schile A. J., Thompson W. H. (2017). J. Chem. Phys..

[cit89] Moskun A. C., Jailaubekov A. E., Bradforth S. E., Tao G., Stratt R. M. (2006). Science.

[cit90] Tao G., Stratt R. M. (2006). J. Chem. Phys..

[cit91] Hunt P. A., Ashworth C. R., Matthews R. P. (2015). Chem. Soc. Rev..

