# Supplementary material for: Solvation dynamics in polar solvents and imidazolium ionic liquids: failure of linear response approximations
Source: Phys Chem Chem Phys. 2018 Jan 29;20(7):5246–55. doi: 10.1039/c7cp07052g (PMC5815284; doi:10.1039/c7cp07052g)
Supplement: Supplementary file 1 [file CP-020-C7CP07052G-s001.pdf]

Electronic Supplementary Material (ESI) for Physical Chemistry Chemical Physics.
This journal is © the Owner Societies 2018

Supporting information for:

Solvation dynamics in polar solvents and imidazolium ionic liquids: Failure of linear response approximations

Esther Heid and Christian Schröder

University of Vienna, Faculty of Chemistry, Department of Computational Biological Chemistry, Währingerstraße 19, A-1090 Vienna, Austria

christian.schroeder@univie.ac.at

Contents

1 Linear-linear and log-linear plots of the solvation response S1
2 Effect of sampling and forcefield S4
3 Higher-order correlation functions S5
4 Partial correlation functions S7
5 Partial higher-order correlation functions S8
6 Higher-order corrections to Gaussian statistics S10
7 Radial distribution functions and coordination numbers S13

1 Linear-linear and log-linear plots of the solvation response

Linar-linear plots of the solvation response in ACN, MeOH and 2-PrOH are shown in Fig. S1. Logarithmic-linear plots of the solvation response of both chromophores in all five solvents are shown in Fig. S2.

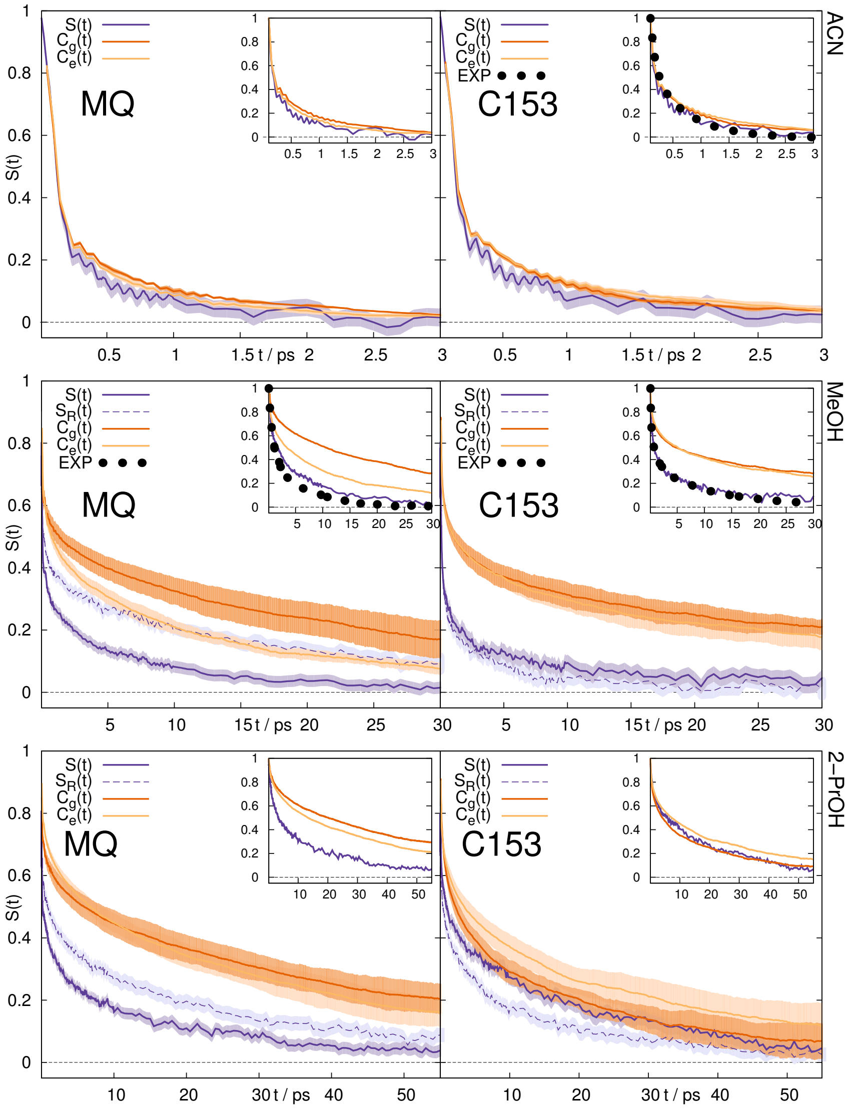

Figure S1: Stokes shift relaxation function after excitation, $S(t)$, after deexcitation, $S_R(t)$, and time correlation functions $C_g(t)$ and $C_e(t)$ in ground and excited state in acetonitrile (top), methanol (middle) and 2-propanol (bottom) for MQ (left) and C153 (right). The colored area corresponds to a 95% confidence interval. Experimental data (labeled EXP) from Ref. [1] and [2].

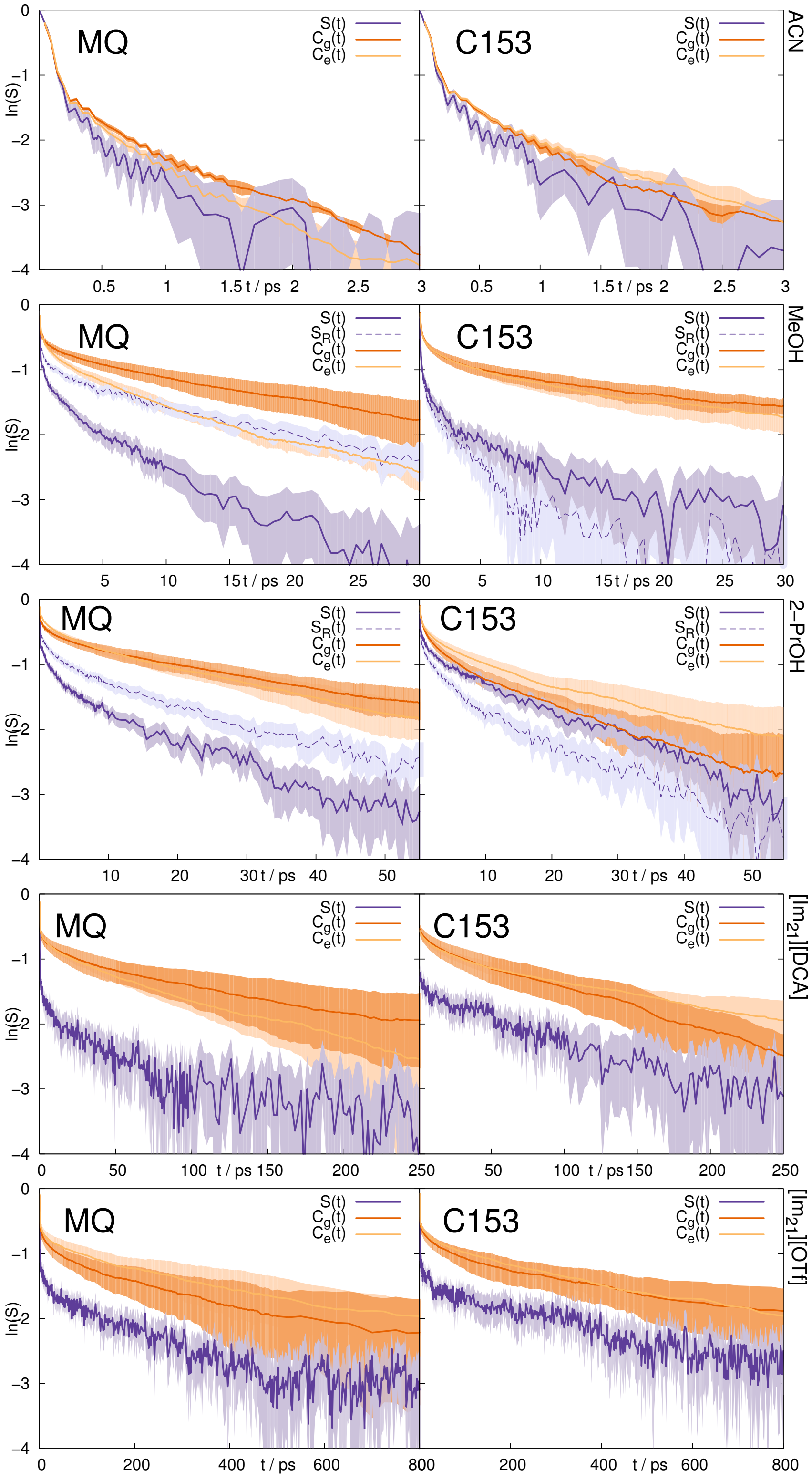

Figure S2: Logarithm of the Stokes shift relaxation function after excitation, $S(t)$, after deexcitation, $S_R(t)$, and time correlation functions $C_g(t)$ and $C_e(t)$ in ground and excited state in acetonitrile (top), methanol (second), 2-propanol (third), $[Im_{21}][DCA]$ (fourth) and $[Im_{21}][OTf]$ (bottom) for MQ (left) and C153 (right). The colored area corresponds to a 95% confidence interval.

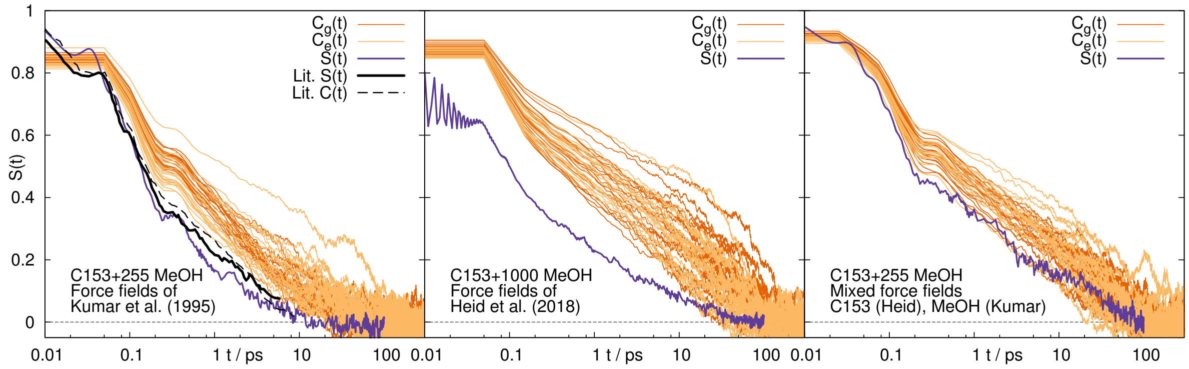

Figure S3: Comparison of $C(t)$ and $S(t)$ using forcefields from Ref. [3] (left), this study (middle) or a mixed description (right), where each correlation function $C(t)$ is obtained from a single short trajectory of 1 ns length.

2 Effect of sampling and forcefield

In Ref. [3] Kumar and Maroncelli find that the equilibrium and nonequilibrium simulation of C153 in 255 molecules of MeOH yields $S(t) \simeq C(t)$. However, the small number of solvent molecules, as well as the short simulation time (1 ns for the equilibrium simulations) lead to an insufficient sampling of configurations. Fig. S3 shows 20 individual equilibrium simulations of 1 ns length in the ground and excited state, as well as the true nonequilibrium response. For the left panel, forcefields for C153 and MeOH, as well as the general simulation setup (rigid molecules, coarse-grained solvent, no polarizability) correspond to Ref. [3], where the black lines correspond to data taken directly from literature. It is evident that depending on the initial configuration of the system, different findings can be obtained, where some of the obtained correlation functions $C(t)$ equal $S(t)$, but others do not. Clearly, 1 ns of simulation time is not sufficient to sample all important configurations, so that repeating the simulation (as shown for 20 different initial coordinates) leads to different conclusions. A similar analysis, namely the use of short trajectories of 1 ns length to describe the equilibrium response, also leads to large uncertainties in $C(t)$ for the force fields and setup used in the main article (middle panel of Fig. S3). Nevertheless, even the fastest equilibrium response does not equal $S(t)$, in contrast to the simulation setup of Ref. [3]. The equilibrium and nonequilibrium results differ to a larger extent for the flexible C153 in atomistic, polarizable MeOH than for the rigid C153 in rigid, coarse-grained MeOH. To detect whether this increased discrepancy stems from the solute or solvent force field, we conducted also simulations of flexible C153 (parametrization used in this study) in the rigid, coarse-grained MeOH employed in Ref. [3] (right panel in Fig. S3). We find that a high-level solvent force field leads to larger differences between $C(t)$ and $S(t)$.

3 Higher-order correlation functions

According to Wick's theorem [4], higher-order correlation functions are multiples of the first-order correlation $\langle \delta\Delta U(0)\delta\Delta U(t)\rangle_e$

$$\langle \delta\Delta U(0)^{2n+1}\delta\Delta U(t)\rangle_e = \frac{(2n+1)!}{n!2^n}\langle \delta\Delta U(0)\delta\Delta U(t)\rangle_e \cdot \langle \delta\Delta U(0)^2\rangle_e^n \tag{1}$$

for odd orders, and zero for even orders. $C_e(t)$ and its higher order factorizations are plotted in Fig. S4 for MQ and C153 in ACN, MeOH, [Im_{21}][DCA] and [Im_{21}][OTf]. The corresponding figure for 2-PrOH can be found in the main article. For both chromophores in ACN, MeOH, [Im_{21}][DCA] and [Im_{21}][OTf], Wick's theorem applies, so that no failure of linear response theory can be deduced from this analysis.

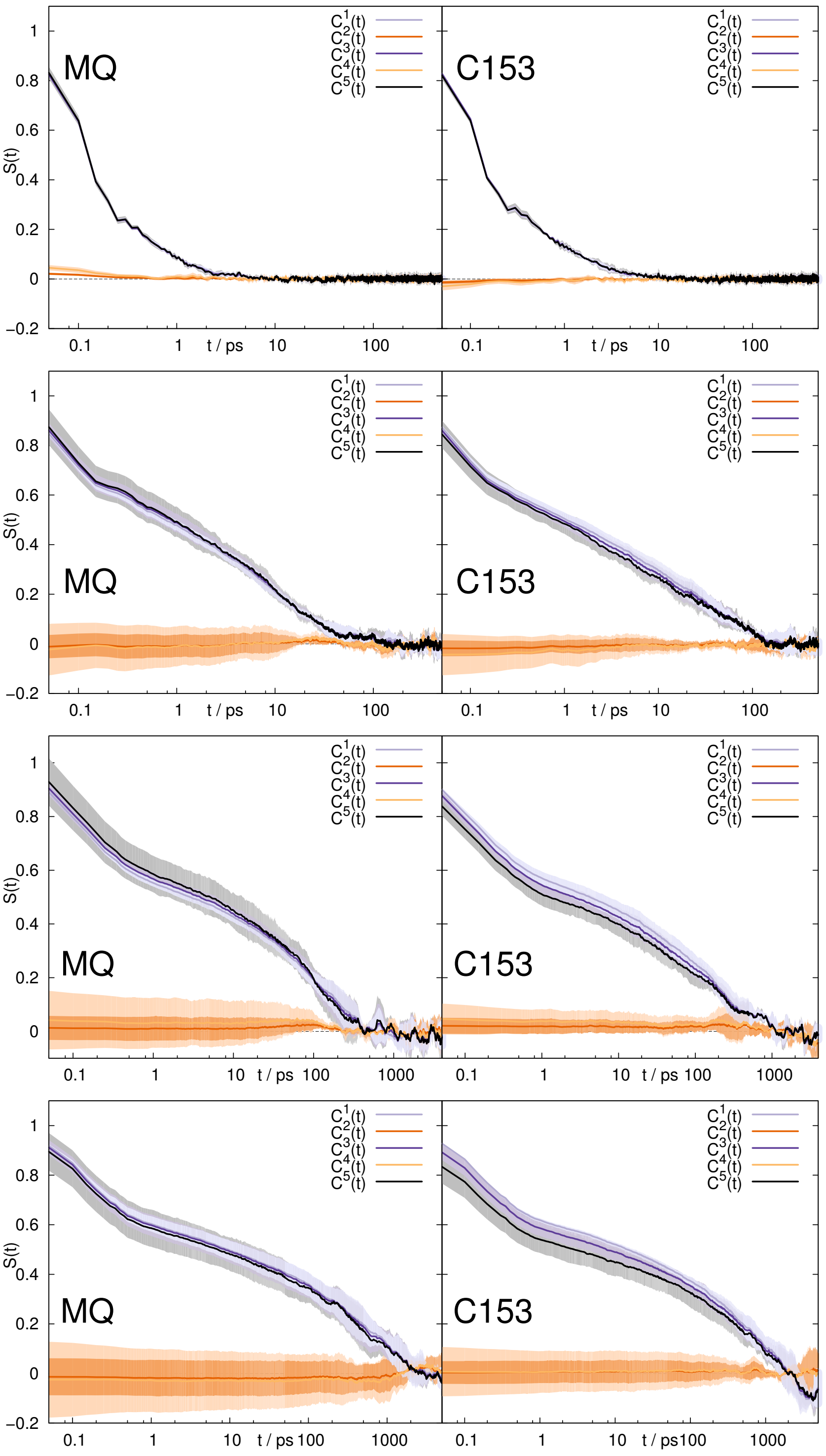

Figure S4: Higher order correlation functions C^n, where $C^n = \frac{\langle \delta\Delta U(0)^n \delta\Delta U(t)\rangle_e}{a_n \langle \delta\Delta U(0)^{n+1}\rangle_e}$ and $a_1 = 1$, $a_2 = 1.6$, $a_3 = 3$, $a_4 = 6.4$ and $a_5 = 15$ according to Eq. (1) after rearrangement. Solvents: acetonitrile (top panel), methanol (second panel), [Im_{21}][DCA] (third panel) and [Im_{21}][OTf] (bottom panel). The colored area corresponds to a 95% confidence interval.

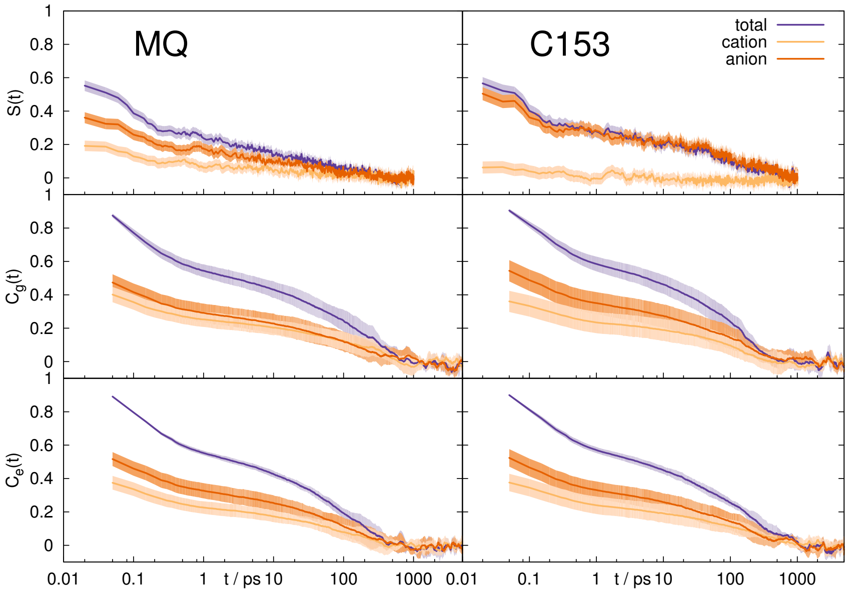

Figure S5: Contributions from cations and anions to the Stokes shift relaxation function $S(t)$ (top panel) and time correlation functions C_g (middle panel) and C_e (bottom panel) in ground and excited state in $[Im_{21}][DCA]$ for MQ (left) and C153 (right).The colored area corresponds to a 95% confidence interval.

4 Partial correlation functions

Fig. S5 shows the partial correlation functions

$$C_Z(t) = \frac{\langle \delta\Delta U_Z(0)\delta\Delta U(t)\rangle}{\langle \delta\Delta U(0)^2\rangle} = \frac{\langle \delta\Delta U(0)\delta\Delta U_Z(t)\rangle}{\langle \delta\Delta U(0)^2\rangle} \tag{2}$$

as well as the true nonequilibrium contributions

$$S_Z(t) = \frac{\overline{\Delta U_Z(t)} - \overline{\Delta U_Z(\infty)}}{\overline{\Delta U(0)} - \overline{\Delta U(\infty)}} \tag{3}$$

from cation ($Z = C$) movement and anion ($Z = A$) movement to the overall solvation response in $[Im_{21}][DCA]$. The corresponding figure for $[Im_{21}][OTf]$ is printed in the main article. The top panel of Fig. S5 shows $S_A(t)$ and $S_C(t)$, where it is clear that the anions contribute more to $S(t)$, especially for C153 as chromophore. The partial correlation functions, however only partly capture this behavior in the case of MQ and fail to describe the correct partial relaxation for C153. Thus, the use of LRT to predict individual contributions to the overall functions, should be carefully tested for each system in use, as it may lead to quantitatively wrong results in some cases.

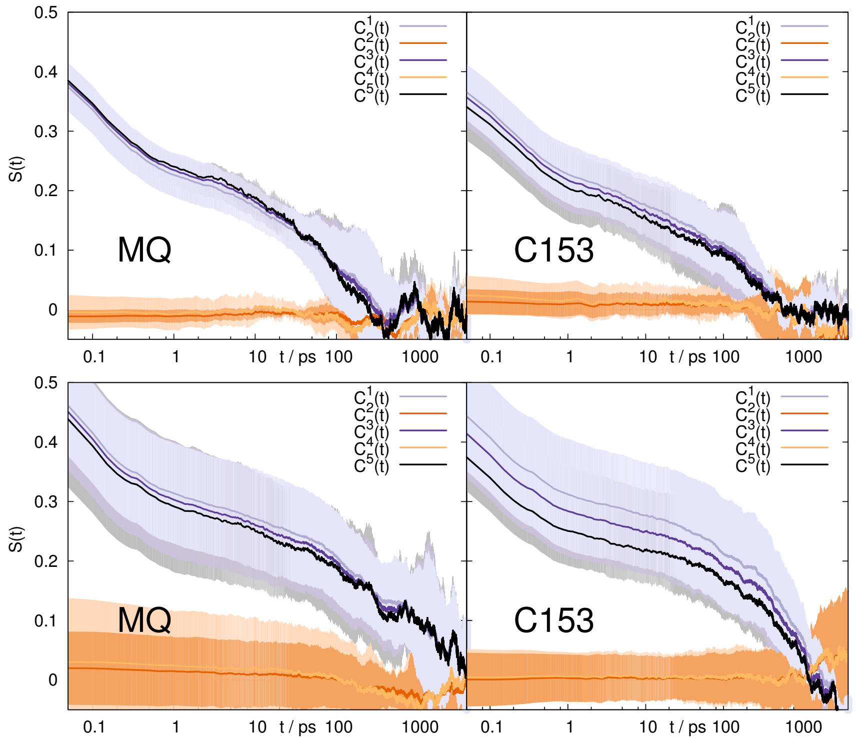

Figure S6: Higher-order correlation functions from cation contributions C_C to the time correlation functions of MQ (left) and C153 (right) in [Im$_{21}$][DCA] (top) and [Im$_{21}$][OTf] (bottom). Definition of C^n as in Fig. S4. The colored area corresponds to a 95% confidence interval.

5 Partial higher-order correlation functions

As explained above, the calculation of higher-order correlation functions and comparison to the first-order correlation function is often used to test the Gaussian statistics of a system. This is also possible for partial correlation functions, so that we calculated the higher-order correlation for $C_C(t)$ and $C_A(t)$, shown in Fig. S6 and S7. The relation inferred from Eqn. 1 holds quite well, although the large confidence interval for [Im$_{21}$][OTf] prevent a detailed analysis. A much longer simulation would be needed to reduce the uncertainty. However, the wrong representation of cationic and anionic contributions to the overall function of both [Im$_{21}$][DCA] and [Im$_{21}$][OTf], as presented in the previous section, does not result from the failure of Wick's theorem in this system.

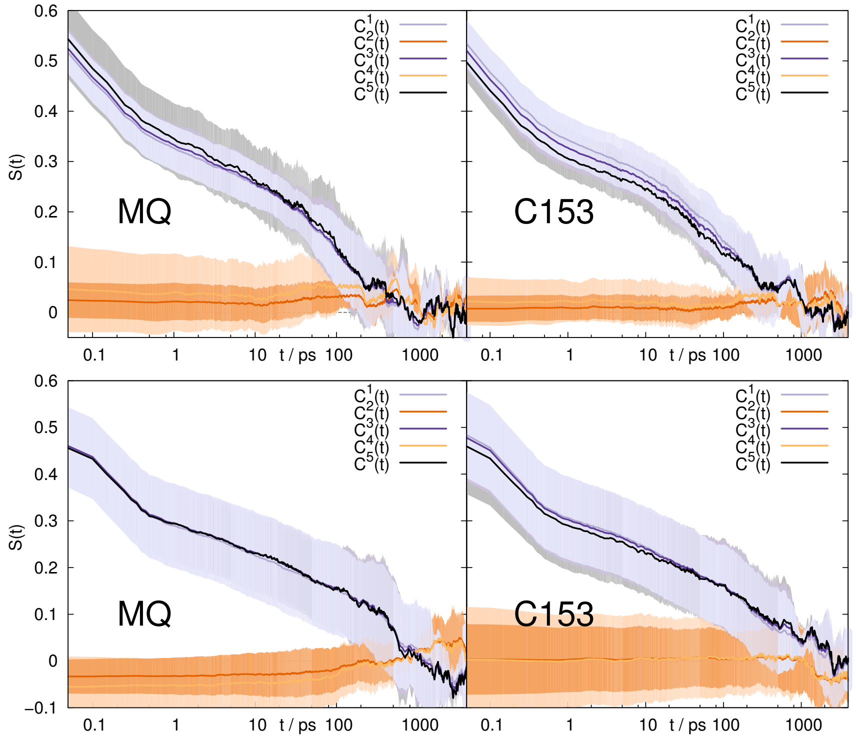

Figure S7: Higher-order correlation functions from anion contributions C_A to the time correlation functions of MQ (left) and C153 (right) in [Im$_{21}$][DCA] (top) and [Im$_{21}$][OTf] (bottom). Definition of C^n as in Fig. S4. The colored area corresponds to a 95% confidence interval.

6 Higher-order corrections to Gaussian statistics

The authors of Ref. [5] suggested to apply corrections to $C(t)$ that result from deviations of Wick‘s theorem.

$$C_e^{corr} \simeq \frac{1}{3k_BT}\sum_{n=0}^{2}\frac{n!2^n}{(2n+1)!}\frac{\langle\delta\Delta U(0)^{2n+1}\delta\Delta U(t)\rangle_e}{\langle\delta\Delta U(0)^2\rangle_e^n} + \frac{1}{2(k_BT)^2}\langle\delta\Delta U(0)\delta\Delta U(t)^2\rangle_e \qquad (4)$$

The results for MQ and C153 in 2-PrOH, where large deviations from Eqn. 1 were found, are shown in the main article. For all other solvents, where deviations from Wick‘s theorem are very small, of course the resulting corrections are very small, as depicted in Fig. S8. In Ref. [5], a small artificial solute was employed to test the usefulness of such corrections. We therefore also applied the corrections to formerly conducted simulations of artificial excitations in benzene [6], where we observed deviations from Eqn. 1. The corresponding excitations are shown in Fig. S9, where we chose the dipole and quadrupole increase and decrease, to test whether Eqn. 4 provides any improvement over $C_e(t)$. As depicted in Fig. S10, for an increase in dipole or quadrupole moment (analogous to what we find in C153), the corrections are somewhat helpful, although for the quadrupole increase the corrections moves the curve in the right direction, but too far. For a decrease in dipole or quadrupole moment (similar to MQ), no improvement was found for $C_{corr}(t)$ over $C_e(t)$. It is interesting that for MQ and C153 in 2-PrOH, we find similar results, were only for C153 (increasing dipole upon excitation) $C_{corr}(t)$ showed a better fit to $S(t)$ than $C_e(t)$.

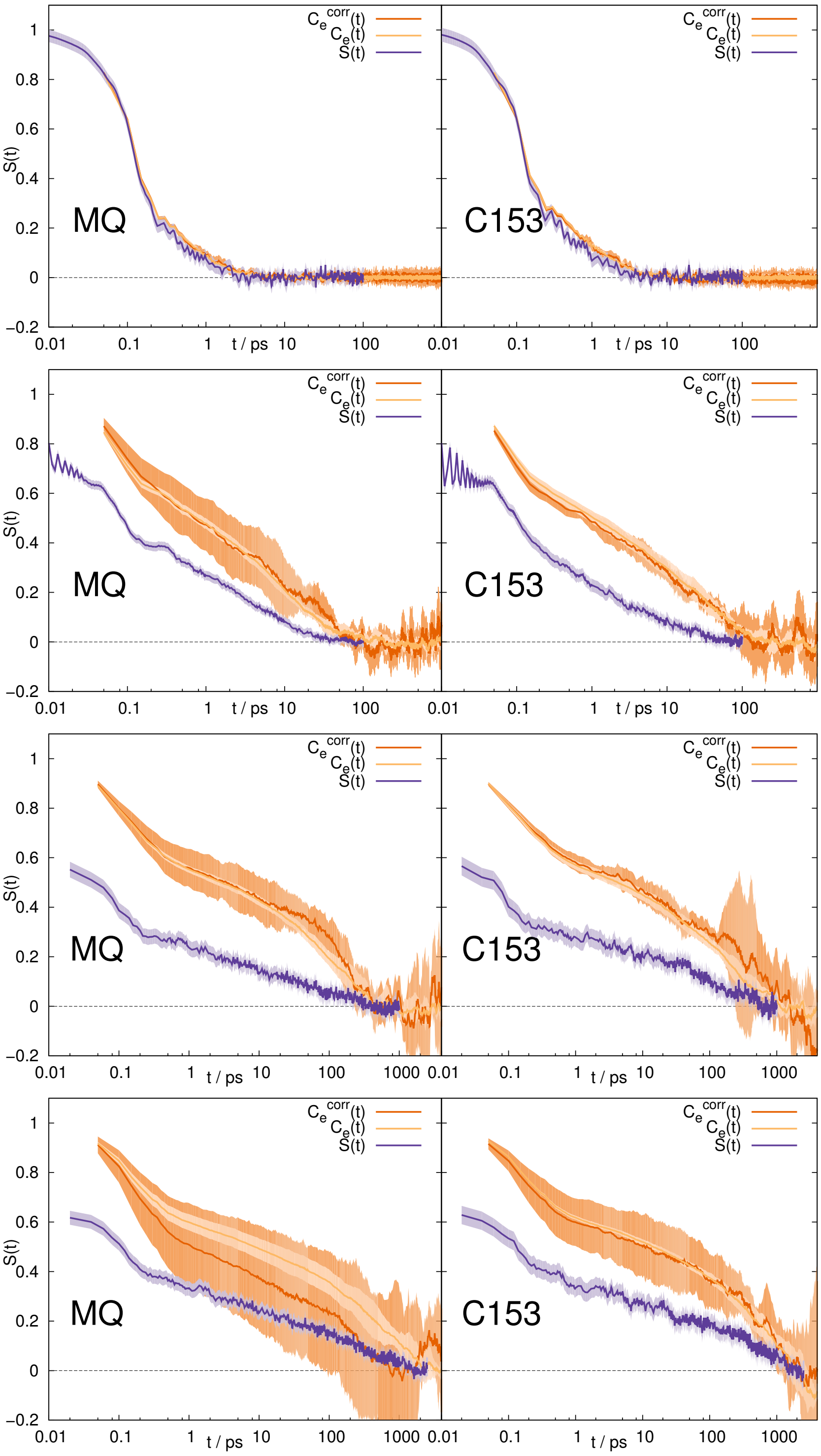

Figure S8: Normalized correction C_e^{corr} to the time correlation function C_e up to the order of three in ACN (top), MeOH (second), [C_2mim][DCA] (third) and [C_2mim][OTf] (bottom) for MQ (right) and C153 (left). The colored area corresponds to a 95% confidence interval.

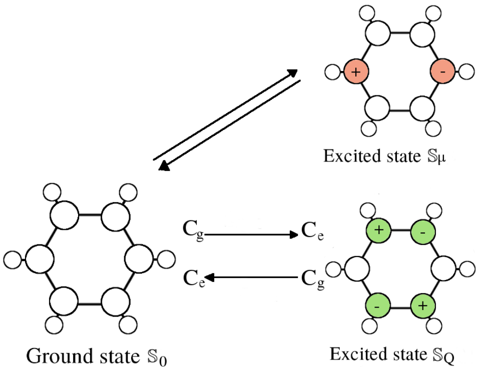

Figure S9: Benzene excitation, adapted from Ref. [6].

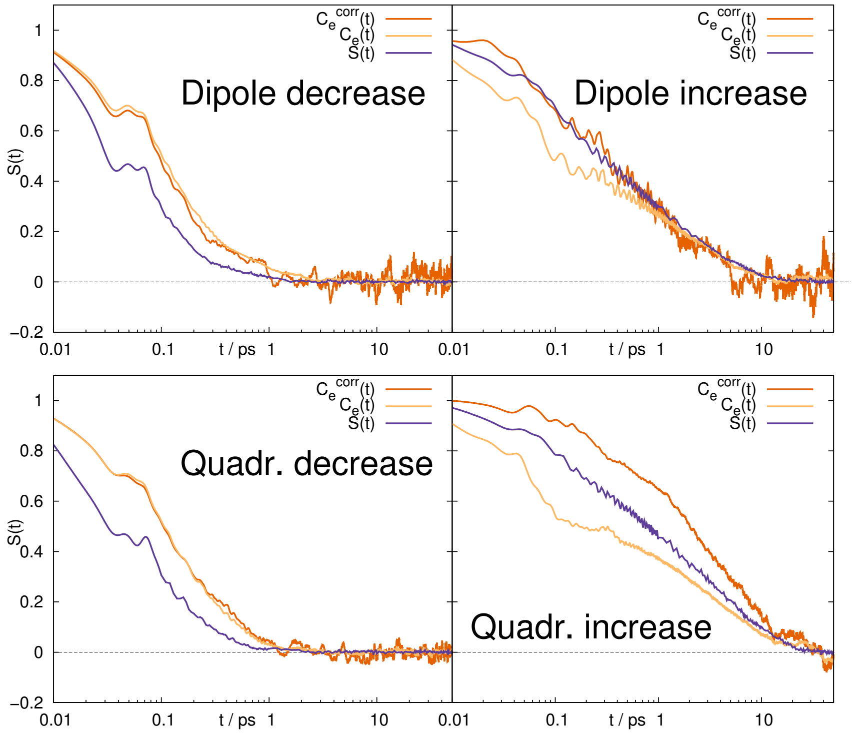

Figure S10: Normalized correction C_e^{corr} to the time correlation function C_e up to the order of five in MeOH for changes in the dipole moment (top) or the quadrupole moment (bottom) in an artificial benzene-like solute. The weakening of the dipole moment (top, right) loosely resembles MQ, whereas the strengthening of dipole moment (top, left) resembles C153.

7 Radial distribution functions and coordination numbers

Fig. S11 shows the radial distribution functions of solvent molecules around MQ and C153. In MeOH and 2-PrOH, quite large restructuring occurs around MQ after excitation, whereas nearly no structure changes occur around C153. In [Im_{21}][DCA] there is nearly no change for both chromophores if all solvent molecules are taken into account. In contrast, the contributions from cations and anions change to some extent around MQ and C153. Fig. S12 shows the corresponding cation and anion coordination numbers, and their evolution with time after excitation. Note that the first shell, obtained via Voronoi tessellation, is quite large, so that more than the first peak in the radial distribution functions contributes to the first shell. The corresponding figures for both chromophores in [C_2mim][OTf] are printed in the main article.

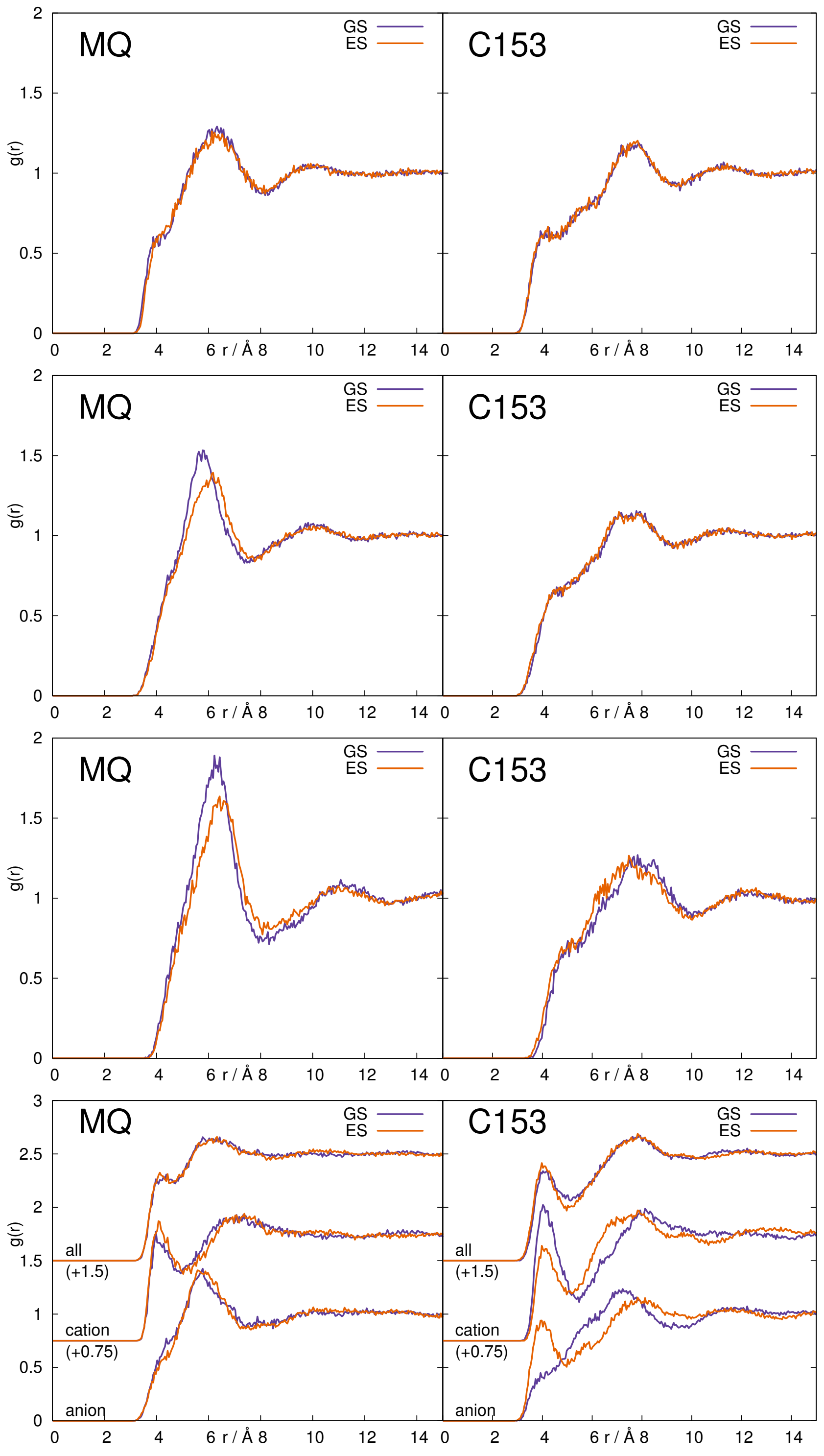

Figure S11: Radial distribution functions of solvent molecules around the central solute MQ (left), or C153 (right). Solvents: acetonitrile (top panel), methanol (second panel), 2-propanol (third panel) and $[Im_{21}][DCA]$ (bottom panel).

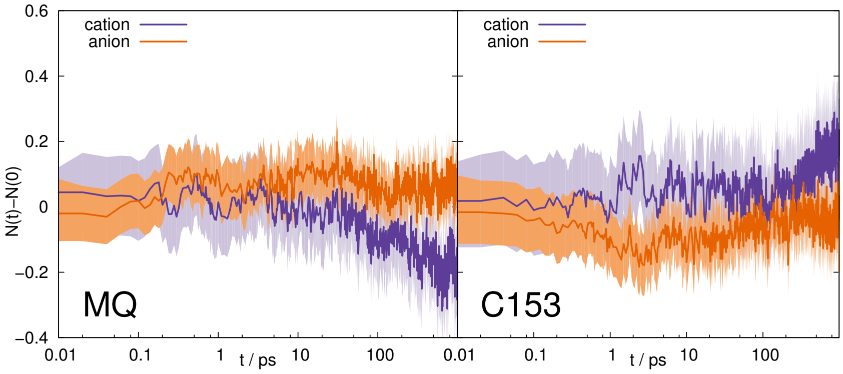

Figure S12: Change in number of first shell ions around the central solute MQ (left), or C153 (right) in [Im_{21}][DCA]. The colored area corresponds to a 95% confidence interval.

References

[1] M. Sajadi, M. Weinberger, H.-A. Wagenknecht, and N. P. Ernsting. "Polar Solvation Dynamics in Water and Methanol: Search for Molecularity." *Phys. Chem. Chem. Phys.*, **13**(2011), 17768.

[2] M. L. Horng, J. A. Gardecki, A. Papazyan, and M. Maroncelli. "Subpicosecond Measurements of Polar Solvation Dynamics: Coumarin 153 Revisited." *J. Phys. Chem.*, **99**(1995), 17311.

[3] P. V. Kumar and M. Maroncelli. "Polar Solvation Dynamics of Polyatomic Solutes: Simulation Studies in Acetonitrile and Methanol." *J. Chem. Phys.*, **103**(1995), 3038.

[4] G. C. Wick. "The evaluation of the collision matrix." *Phys. Rev.*, **80**(1950), 268.

[5] A. J. Schile and W. H. Thompson. "Test for, origins of, and corrections to non-gaussian statistics. the dipole flip model." *J. Chem. Phys.*, **146**(2017), 154109.

[6] E. Heid, W. Moser, and C. Schröder. "On the Validity of Linear Response Approximations Regarding the Solvation Dynamics of Polyatomic Solutes." *Phys. Chem. Chem. Phys.*, **19**(2017), 10940.
